# Factors associated with low-level viremia in people living with HIV: A 10-year retrospective study in South Korea

**DOI:** 10.1371/journal.pone.0350391

**Published:** 2026-06-16

**Authors:** Eunmi Yang, Subin Kim, Doh Hee Kim, Mi Young Ahn, Jae-Phil Choi

**Affiliations:** 1 Division of Infectious Diseases, Hallym University Sacred Heart Hospital, Hallym University College of Medicine, Anyang, South Korea; 2 Division of Infectious Disease, Seoul Medical Center, Seoul, South Korea; 3 Department of Research Institute, Seoul Medical Center, Seoul, South Korea; Katholieke Universiteit Leuven Rega Institute for Medical Research, BELGIUM

## Abstract

**Purpose:**

The goal of antiretroviral therapy is to achieve and sustain the suppression of human immunodeficiency virus (HIV) viral load. In this study, we aimed to identify risk factors for low-level viremia (LLV) and examine their association with clinical outcomes in South Korea.

**Methods:**

We retrospectively reviewed the medical records of patients with human immunodeficiency virus infection registered at Seoul Medical Center and Hallym University Sacred Heart Hospital between 2014 and 2023. LLV was defined as at least two consecutive HIV RNA measurements of 40–199 copies/mL taken more than 4 weeks apart. Patients with LLV were compared with those who maintained virological suppression (viral load < 40 copies/mL).

**Results:**

Of the 381 patients included in the analysis, 15 (3.94%) experienced LLV. Compared with patients who maintained virological suppression, patients with LLV more frequently had an initial viral load ≥ 500,000 copies/mL (*P* < 0.01). No significant differences were observed between the groups in the rates of virological rebound, new-onset acquired immune deficiency syndrome–defining conditions, or mortality. Multivariable logistic regression identified an initial viral load ≥ 500,000 copies/mL as an independent risk factor for LLV (adjusted odds ratio, 4.735; 95% confidence interval, 1.505–14.897).

**Conclusion:**

A high viral load was a significant risk factor for LLV. Large multicenter studies are required to further investigate risk factors and clinical implications of LLV in people living with HIV.

## Introduction

Antiretroviral therapy (ART) reduces the morbidity and mortality of individuals infected with the human immunodeficiency virus (HIV) [1]. Its primary goals are virological suppression, restoration and preservation of immune function, reduction of HIV-related complications, and prevention of transmission [2]. Although most individuals achieve sustained virologic suppression with ART, transient and persistent increases in the viral load are commonly observed. A transient increase followed by suppression is referred to as a blip, whereas persistent detectable viremia is termed low-level viremia (LLV) [2]. Although virological blips are not typically associated with treatment failure, the clinical significance of persistent LLV remains uncertain [3,4]. Some studies suggest that LLV levels between 50 and 199 copies/mL are not associated with virological failure, whereas others report an increased risk of failure, resistance, and mortality [5–8].

The prevalence of LLV varies by region. Crespo-Bermejo et al. [5] reported a higher prevalence in Africa (22.3%) than in Asia (15.6%). Zhang et al. [6] reported an LLV prevalence of 10.6% in China and identified baseline viral load, CD4 count, and initiation of a protease inhibitor (PI)–based regimen as factors associated with LLV. These regional differences in LLV prevalence may be attributable to variations in HIV subtypes, ART regimens influenced by economic status, and differences in treatment adherence related to public health systems.

In Korea, approximately 15,000 people living with HIV (PLWH) are registered, with approximately 1,000 new cases reported annually. However, no studies have investigated LLV in Korean cohorts. Therefore, in this study, we aimed to assess the current status of LLV in South Korea and evaluate the risk factors and clinical outcomes associated with LLV.

## Materials and methods

### Study population

This multicenter retrospective cohort study was conducted at Seoul Medical Center and Hallym University Sacred Heart Hospital in South Korea. We reviewed the medical records of PLWH who received ART between January 2014 and December 2023. The inclusion criterion was an age of at least 16 years at the time of ART initiation. The exclusion criteria were as follows: patients within 6 months of initiating ART, those with an observation period <6 months, and those with poor drug adherence. Adherence was assessed through interviews conducted during outpatient visits every 3–6 months and was considered poor if patients discontinued their medication independently, missed doses for >7 days, or had leftover medication for >7 days, consistent with the criteria used in a previous study [7]. Patients who received ART at another hospital and had unknown initial CD4 count or initial acquired immune deficiency syndrome (AIDS)–defining conditions were also excluded from the study.

Baseline characteristics included demographics, country of birth, age, underlying disease, Charlson Comorbidity Index, hepatitis B virus infection, and hepatitis C virus co-infection at ART initiation. Initial AIDS-defining conditions, ART regimens, and resistance-associated mutations at screening were compared between patients with and without viral suppression. The Charlson Comorbidity Index was used to assess the severity of comorbidities, excluding AIDS from the calculation [8]. Clinical outcomes included virological rebound, new-onset AIDS-defining conditions, emergence of resistance-associated mutations, and all-cause mortality during the study period.

### Data collection

Access to medical records for research began on January 3, 2023, at Seoul Medical Center, and on February 25, 2025, at Hallym University Sacred Heart Hospital. During data collection, all personally identifiable information was removed, and the authors had no access to such information at any stage, either during or after the data collection.

### Definition

The U.S. Department of Health and Human Services defines LLV as HIV RNA levels above the lower limit of detection (LLOD) and < 200 copies/mL. In South Korea, most hospitals use assays with an LLOD of 20–40 copies/mL [2]. Accordingly, we defined LLV as at least two consecutive viral measurements between 40 and 199 copies/mL taken at least 4 weeks apart. Viral load was monitored every 3–6 months during outpatient visits. Patients with confirmed LLV at any time during the study period were assigned to the LLV group. Virologic suppression was defined as < 40 copies/mL, and virologic rebound was defined as confirmed HIV RNA levels ≥ 200 copies/mL following virologic suppression. Patients who consistently maintained virological suppression throughout the study period were categorized into the no-LLV group. AIDS-defining conditions and opportunistic infections were classified according to the Centers for Disease Control and Prevention Classification of HIV-related opportunistic illnesses [9]. Drug resistance-associated mutations were assessed by polymerase chain reaction amplification and direct sequencing of the protease, reverse transcriptase, and integrase genes, followed by comparison with the GenBank reference sequence (accession number K03455.1) to identify resistance-associated mutations.

### Statistical analysis

Patients with LLV were compared with those who maintained virological suppression. Categorical variables were analyzed using the chi-square test or Fisher’s exact test, and continuous variables were analyzed using the Mann–Whitney *U* test or Student’s *t*-test. Univariable and multivariable logistic regression models were used to identify risk factors for LLV. All statistical analyses were performed using SPSS software (version 29.0; IBM Corp., Armonk, NY, USA). Statistical significance was set at *P* < 0.05.

### Ethics statement

This study was reviewed and approved by the Institutional Review Board of Seoul Medical Center (SEOUL 2022-12-003) and Hallym University Sacred Heart Hospital (HALLYM 2025-01-003). The requirement for informed consent was waived by the Institutional Review Board.

## Results

### Baseline characteristics

Data were collected from 863 patients who received ART between January 2014 and December 2023 (S1 Fig). A total of 61 patients were excluded because of poor drug adherence, and 301 patients were excluded because they had initiated ART within 6 months or had an observation period of <6 months. Additionally, 120 patients were excluded owing to missing initial CD4 count data. During the study period, 15 patients (3.94%) experienced LLV, whereas 366 patients maintained virological suppression. The duration of LLV was calculated from the first documented LLV episode to the last recorded LLV measurement, with a median duration of 189 days.

Of the study patients, 90% were men and 76.9% were born in Korea. Baseline characteristics of patients in the LLV and no-LLV groups are presented in Table 1. Chronic obstructive pulmonary disease (COPD) was observed only in the LLV group and not in the no-LLV group (*P* = 0.04). Furthermore, an initial viral load ≥ 500,000 copies/mL at ART initiation was significantly more common in the LLV group (*P* < 0.01). No co-infection with hepatitis B or C viruses was observed in the LLV group. There were no statistically significant differences in the baseline Charlson Comorbidity Index scores, CD4 count, initial AIDS-defining conditions, opportunistic infections, ART regimen, or resistance-associated mutations between the two groups. Drug resistance-associated mutations are summarized in Supplementary Table 1 (S1 Table).

**Table 1:** Demographic and clinical characteristics of the study patients.

Patient characteristics	LLV^a^ (n = 15) No. (%)	No LLV (n = 366) No. (%)	*P* value
Male	14 (93.3)	325 (88.8)	>0.99
Age (years), median (IQR)^b^	39 (29.0–52.0)	35 (27.0–46.0)	0.30
Country of birth			
Korea	12 (80.0)	281 (76.8)	>0.99
Underlying disease	8 (53.3)	136 (37.2)	0.21
Hypertension	1 (6.7)	32 (8.7)	>0.99
Diabetes mellitus	3 (20.0)	23 (6.3)	0.07
Congestive heart disease	0	1 (0.3)	>0.99
Coronary artery disease	0	5 (1.4)	>0.99
Cerebrovascular accident	0	8 (2.2)	>0.99
Chronic obstructive pulmonary disease	1 (6.7)	0	0.04
Chronic liver disease	1 (6.7)	14 (3.8)	0.46
Chronic kidney disease	0	5 (1.4)	>0.99
Solid cancer	0	8 (2.2)	>0.99
Hematologic malignancy	1 (6.7)	2 (0.5)	0.11
Charlson Comorbidity Index, median (IQR)	0 (0–2)	0 (0–0)	0.10
Co-infection			
Hepatitis B virus	0	14/364 (3.8)	>0.99
Hepatitis C virus	0	8/356 (2.2)	>0.99
Initial CD4 count (cells/μL), median (IQR)^c^	136 (87–283)	264.5 (95–442)	0.14
Initial VL (log_10_ copies/mL), median (IQR)^d^	5.0 (3.9–6.0)	4.6 (3.9–5.2)	0.12
Initial VL ≥ 500,000 copies/ml	6 (40)	39 (10.7)	<0.01
AIDS-defining conditions	4 (26.7)	78 (21.3)	0.54
Opportunistic infections	2 (13.3)	75 (20.5)	0.75
Initial ART regimen			
NNRTI-based	0	21 (5.7)	>0.99
PI-based	3 (20.0)	69 (18.9)	>0.99
INSTI-based	12 (80.0)	274 (74.9)	0.77
Resistance-associated mutation	6/9 (66.7)	63/148 (42.6)	0.18
NRTI	0/9	15/148 (10.1)	0.60
NNRTI	3/9 (33.3)	23/148 (15.5)	0.17
PI	3/9 (33.3)	38/148 (25.7)	0.70
INSTI	2/9 (22.2)	10/148 (6.8)	0.14

IQR, interquartile range; VL: viral load; NNRTI: non-nucleoside reverse transcriptase inhibitor; INSTI: integrase strand transfer inhibitor; NRTI: nucleoside reverse transcriptase inhibitor.

^a^Two consecutive VL measurements of 40–199 copies/mL, at least 4 weeks apart.

^b^Age at ART initiation.

^c, d^CD4 count and viral load at ART initiation.

### Clinical outcomes

Clinical outcomes of the patients are summarized in Table 2. At the end of the study period, 96.1% of the patients received an integrase strand transfer inhibitor (INSTI)–based regimen, 3.41% received a PI-based regimen, and 0.5% received a non-nucleoside reverse transcriptase inhibitor (NNRTI)–based regimen. No new-onset AIDS-defining conditions, opportunistic infections, or all-cause deaths were observed in the LLV group. The median CD4 count at the end of the study period did not differ between the two groups. One patient in the LLV group experienced a virological rebound after suppression. At the time of LLV detection, 13 patients were on an INSTI-based regimen, and two were on a PI-based regimen.

**Table 2:** Clinical outcomes of the study patients.

	LLV (n = 15) No. (%)	No LLV (n = 366) No. (%)	*P* value
Virologic rebound^a^	1 (6.7)	14 (3.8)	0.46
AIDS-defining conditions	0	10 (2.7)	>0.99
Opportunistic infection	0	8 (2.2)	>0.99
Final CD4 count, median (IQR)	519 (367.0–674.3)	620 (432.2–820.3)	0.15
All-cause death	0	4 (1.1)	>0.99

^a^After virologic suppression, confirmed HIV RNA level ≥ 200.

Fourteen cases of virologic rebound were observed in the no-LLV group. Among patients with virological rebound, new-onset resistance-associated mutations were observed in two patients (Patient 1: V106I and L10I; Patient 2: L10I and E157Q).

### Risk factors associated with low-level viremia

Multivariable analysis was performed to evaluate whether these factors were independently associated with LLV (Table 3). Potential confounding variables were selected based on their biological and clinical relevance to viremia, with reference to previous LLV studies [10,11]. Age at ART initiation, initial opportunistic infection, initial CD4 count, initial viral load ≥ 500,000 copies/mL, and Charlson Comorbidity Index were included as confounding factors in the multivariable logistic regression model. An initial viral load ≥ 500,000 copies/mL (adjusted odds ratio, 4.735; 95% confidence interval [CI], 1.505–14.897) was an independent risk factor for LLV.

**Table 3:** Factors associated with low-level viremia in multivariable logistic regression.

Characteristics	Univariable analysis	Multivariable analysis^a^	
	OR (95% CI)	OR (95% CI)	*P* value
Age at start of ART	1.006 (0.961–1.054)		
Charlson Comorbidity Index	1.260 (0.692–2.294)		
Initial CD4 count	0.997 (0.994–1.001)	0.997 (0.004–1.001)	0.112
Initial viral load ≥ 500,000 copies/mL	4.627 (1.460–14.667)	4.735 (1.505–14.897)	0.008
Initial opportunistic infection	0.257 (0.050–1.334)	0.255 (0.050–1.303)	0.101

^a^This model fit the data well in terms of discrimination (C-statistic = 0.748) and calibration (Hosmer–Lemeshow goodness-of-fit statistic = 7.83; P = 0.45).

## Discussion

In this retrospective cohort study, LLV occurred in 3.94% of the PLWH. Clinical outcomes, such as virological rebound, new-onset AIDS-defining conditions, and all-cause mortality, did not differ significantly between the LLV and non-LLV groups. In the multivariable logistic regression analysis, an initial viral load ≥ 500,000 copies/mL was identified as a risk factor for LLV.

Regarding the baseline characteristics, the prevalence of diabetes mellitus was higher in the LLV group than in the non-LLV group, although the difference was not statistically significant. Only one patient in the LLV group had COPD, and the sample size was too small for statistical evaluation. Underlying comorbidities may promote chronic inflammation and be associated with LLV, while LLV may, in turn, contribute to the development of comorbid conditions. Tao et al. [12] reported that LLV increases the risk of developing diabetes mellitus. Further studies with larger sample sizes are needed to evaluate the association between combined comorbidities and LLV.

The primary goal of ART is to maintain virological suppression in PLWH. Although most patients achieve this goal, some develop LLV despite receiving appropriate therapy. Several hypotheses have been proposed to explain the mechanisms underlying LLV. The viral reservoir size or clonal expansion of HIV-infected cells has been suggested as the origin of LLV [13,14]. However, few studies have clarified the origins and causes of LLV. In an observational study, Brattgård et al. [10] identified male sex, higher pre-ART viral load, lower pre-ART CD4 count, use of a PI-based regimen, and non-standard ART as risk factors for LLV. In our study, the initial CD4 count was not associated with LLV, whereas a high initial viral load was identified as a risk factor for LLV. This indicates that advanced HIV infection and a larger HIV reservoir may contribute to the development of LLV. Consistent with this, Bachmann et al. [15] reported that LLV is associated with a slower decay of the viral reservoir. Early diagnosis of HIV infection and timely ART initiation may help reduce the incidence of LLV.

Previous studies have reported conflicting results regarding the clinical outcomes of LLV. Laprise et al. [16] reported that persistent LLV levels between 50 and 199 copies/mL increased the risk of virological failure. Other studies have shown that LLV > 200 copies/mL is strongly associated with virological failure and AIDS events/death [17–19]. The viral load that causes virological failure remains unclear; however, the risk of virological failure appears to increase with increasing viral load. In our study, only one case of virological rebound was observed in the LLV group, and there was no statistically significant difference between the LLV and non-LLV groups. This may be attributed to the small sample size and high proportion of patients receiving INSTI-based treatment in our cohort. Previous studies have shown that PI-based regimens and non-standard ART are risk factors for LLV [10]. Approximately 80% of the patients in our study initiated INSTI-based therapy, a proportion higher than that reported in other studies [10,11,17,19]. Recent INSTI-containing regimens have demonstrated high and durable efficacy, along with better tolerability than boosted PI-containing regimens [20–22]. The use of INSTI-based regimens may influence both the incidence and clinical outcomes of LLV. However, further studies are required to clarify this relationship.

Our study has some limitations. Because this was a retrospective study, it was subject to inherent limitations in its interpretation. The small sample size might have influenced the outcomes and introduced statistical uncertainty. Therefore, multicenter studies with longer observation periods are necessary to fully assess the occurrence and implications of LLV. Additionally, drug adherence was assessed through patient interviews, which might have introduced bias and affected the incidence of LLV. Because adherence is essential for maintaining viral suppression and preventing LLV, more accurate assessment methods, such as requesting patients to bring remaining medication, using daily checklists, questionnaires, or measuring therapeutic drug monitoring, are needed. Standardized questionnaires and validated scoring systems should be incorporated into future studies to address these limitations. Finally, given that several studies on LLV have already been reported, the originality of this study may be questioned by some. Nevertheless, despite these limitations, this study is meaningful as it is the first investigation of LLV in South Korea.

In conclusion, a high initial viral load is an independent risk factor for LLV. Clinical failures, such as virologic rebound, AIDS-defining conditions, and death, were not significantly associated with patients with LLV compared with those with virologic suppression. Further multicenter studies are needed to better understand LLV in PLWH in South Korea.

## Supporting information

S1 Fig Flow chart of study population. (TIF)

S1 Table Drug resistance-associated mutations in study patients. (PDF)

S1 Material The dataset generated and analyzed during the current study. (PDF)
