# Supplementary figures and images for: Factors associated with low-level viremia in people living with HIV: A 10-year retrospective study in South Korea
Source: PLoS One. 2026 Jun 16;21(6):e0350391. doi: 10.1371/journal.pone.0350391 (PMC13271519; doi:10.1371/journal.pone.0350391)

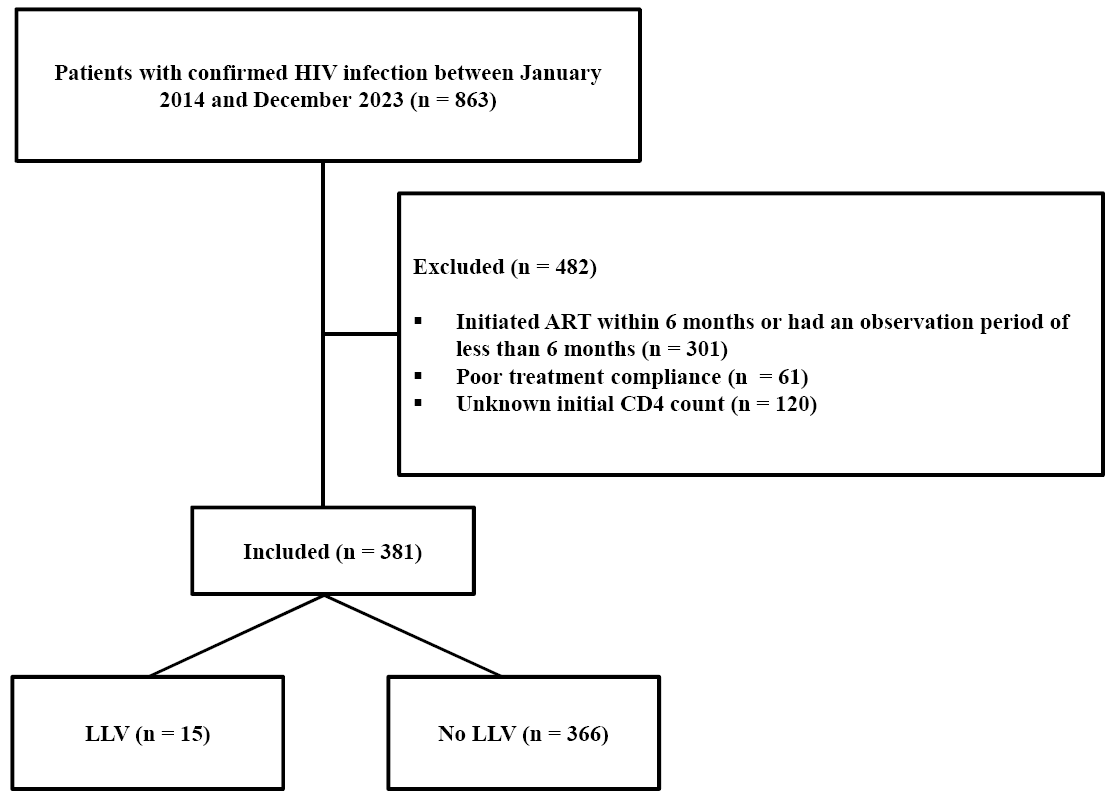

Supplement: S1 Fig — (TIF) [file pone.0350391.s001.tif]
