# Supplementary material for: Factors associated with low-level viremia in people living with HIV: A 10-year retrospective study in South Korea
Source: PLoS One. 2026 Jun 16;21(6):e0350391. doi: 10.1371/journal.pone.0350391 (PMC13271519; doi:10.1371/journal.pone.0350391)
Supplement: S1 Table — (PDF) [file pone.0350391.s002.pdf]

Supplementary Table 1. Drug resistance-associated mutations in study patients

Resistance class	Resistance mutations (number of patients)
INSTI	E157Q (6)
	T97A (2)
	V151VL (1)
	T66I (1)
	E92Q (1)
	N155H (1)
NNRTI	V179D (10)
	E138G (6)
	V106I (4)
	V179E (2)
	V179T (2)
	K103N (2)
	G190A (2)
	A98G (2)
	K101E (1)
	K101Q (1)
	V90I (1)
NRTI	A62V (4)
	T69N (4)
	M184I (3)
	M184V (3)
	M41L (2)
	K219R (2)
	D69N (1)
	V118I (1)
	K219E (1)
	K219Q (1)
	K70T (1)
	L74V (1)
	K65R (1)
PI	L10I (20)
	L10V (12)
	A71V (8)
	A71T (5)
	V11I (2)
	Q58E (2)
	V82S (1)
