# Supplementary material for: Factors associated with low-level viremia in people living with HIV: A 10-year retrospective study in South Korea
Source: PLoS One. 2026 Jun 16;21(6):e0350391. doi: 10.1371/journal.pone.0350391 (PMC13271519; doi:10.1371/journal.pone.0350391)
Supplement: S1 Material — (PDF) [file pone.0350391.s003.pdf]

number	age	sex	nation	height	weight	BMI	LLV	underlygir	HTN	DM	CHD	MI	CVA	COPD	CLD	CKD	solid c	hematol	Charlson	I_INSTI	I_NNRTI	I_PI	I_BIC	I_DTG	ini_lab	ini_wbc	ini_CD4	ini_RNA	ini_HBV	ini_HCV	ini_NRTI_R	ini_INSTI_R
1	75	1	1	160	54	21.1	0	1	0	0	0	0	0	0	0	0	0	0	0	1	0	0	0	0	20161004	5	191	18431	0	0	0	1
2	61	1	1	157	38	15.4	0	1	0	0	0	0	1	0	0	0	0	0	1	1	0	0	0	0	20170104	2	106	610000	0	0		
3	49	0	1				0	0	0	0	0	0	0	0	0	0	0	0	0	1	0	0	0	0	20180410	5.2	79	3123951	1	0	0	0
4	44	1	1	170	60	20.8	0	1	0	0	0	0	1	0	0	0	0	0	3	1	0	0	0	0	20190423	8.2	31	0	0	0	0	0
5	33	1	1				0	0	0	0	0	0	0	0	0	0	0	0	0	1	0	0	0	0	20160418	6.6	619	419	0	0		
6	33	1	1	170	59	20.4	0	1	0	0	0	0	0	0	0	0	0	0	0	1	0	0	0	0	20150317	3.9	369	1017	0	0		
7	49	1	1				0	0	0	0	0	0	0	0	0	0	0	0	0	0	0	1	0	0	20140429	5.9	328	45500	0	0		
8	41	1	1	175.2	72.7	23.7	0	0	0	0	0	0	0	0	0	0	0	0	0	1	0	0	0	0	20140708	7.4	404	63022	0	0		
9	67	1	1				0	1	0	0	0	0	0	0	0	0	0	0	1	0	0	1	0	0	20140916	5.5	436	226	0	0	0	0
10	47	1	1				0	1	0	0	0	0	0	0	0	0	0	0	1	1	0	0	0	0	20140624	6.5	52	202000	0	0		
11	45	1	1	165	66	24.2	0	0	0	0	0	0	0	0	0	0	0	0	0	1	0	0	0	0	20140624	6.3	191	0	0	0		
12	58	1	1				0	1	1	0	0	0	0	0	0	0	0	0	1	1	0	0	0	0	20100901	5.2	22	0	0	0		
13	51	1	1				0	1	1	0	0	0	0	0	0	0	0	0	0	1	0	0	0	0	20110420	7.6	174	0	0	0		
14	43	1	1				0	1	1	1	0	0	0	0	0	0	0	0	1	1	0	0	0	0	20160404	7.4	658	1952	0	0		
15	27	1	1				0	0	0	0	0	0	0	0	0	0	0	0	0	1	0	0	0	0	20110812	7.3	102	1320000	0	0		
16	41	1	1				0	1	0	0	0	0	0	0	0	0	0	0	0	1	0	0	0	0	20020101	5.8	150	0	0	0		
17	52	1	1				1	1	0	1	0	0	0	0	0	0	0	0	2	0	0	1	0	0	20111020	6.9	27	3340000	0	0		
18	23	1	1				0	1	0	0	0	0	0	0	0	0	0	0	0	1	0	0	0	0	20111001	6.9	241	10600	0	0		
19	24	1	1				0	0	0	0	0	0	0	0	0	0	0	0	0	1	0	0	0	0	20160325	6.1	735	22079	0	0	0	0
20	75	1	1	168	76	26.9	0	1	1	1	1	0	0	0	0	1	0	0	4	1	0	0	0	0	20140415	7.6	361	1047	0	0		
21	45	1	1				0	1	0	0	0	1	0	0	1	0	1	0	4	1	0	0	0	0	20120222	6.3	89	33200	0	0		
22	25	1	1				0	0	0	0	0	0	0	0	0	0	0	0	0	1	0	0	0	0	20121214	6.7	388	127484	0	0		
23	27	1	1				0	0	0	0	0	0	0	0	0	0	0	0	0	1	0	0	0	0	20120222	5.8	104	439000	0			
24	46	0	1				0	1	0	0	0	0	0	0	1	0	1	0	3	0	1	0	0	0	20101115	3.7	230	0	0	1		
25	52	1	1				0	0	0	0	0	0	0	0	0	0	0	0	0	0	0	1	0	0	20120101	6.5	214	990000	0	0		
26	36	1	1				0	0	0	0	0	0	0	0	0	0	0	0	0	0	0	1	0	0	20120720	5.9	370	45009	0	0		
27	58	1	1	169.9	69	23.9	0	1	1	0	0	0	0	0	0	0	0	0	0	1	0	0	1	0	20191217	4.9	29	266256	0	0	0	0
28	18	1	1				0	0	0	0	0	0	0	0	0	0	0	0	0	0	0	1	0	0	20120927	8.6	338	14335	0	0		
29	52	1	1				0	1	0	0	0	0	0	0	0	0	0	0	0	0	0	1	0	0	20140415	4.9	120	29885	0	0		
30	33	1	1				0	1	0	0	0	0	0	0	0	0	0	0	0	0	0	1	0	0	20121112	6.5	227	0	0	0		
31	54	1	1				0	1	0	0	0	0	0	0	0	0	0	0	0	0	0	1	0	0	20120201	8.4	35	0	0	0		
32	35	1	1				0	1	1	0	0	0	0	0	0	0	0	0	0	1	0	0	0	0	20151231	5.6	611	11869	0	0		
33	43	1	1				0	1	0	0	0	0	0	0	0	0	0	0	0	0	1	0	0	0	20121205	7.7	155	0	0	0		
34	44	1	1				0	1	0	0	0	0	0	0	0	0	1	0	2	0	0	1	0	0	20121122	5.3	128	0	0	0		
35	34	1	1				0	1	0	0	0	0	0	0	0	0	0	0	0	0	0	1	0	0	20130116	4.4	12	408161	0	0		
36	41	1	1				0	1	1	0	0	0	0	0	0	0	0	0	0	0	0	1	0	0	20130123	5.7	251	46830	0	0		
37	40	1	1				0	0	0	0	0	0	0	0	0	0	0	0	0	1	0	0	0	0	20151201	7.1	538	103225	0	0		
38	51	1	1				0	0	0	0	0	0	0	0	0	0	0	0	0	1	0	0	0	0	20130222	6.6	276	252671	0	0		
39	44	1	1				0	1	0	0	0	0	0	0	0	0	1	0	0	1	0	0	0	0	20140929	5.6	311	19949	0	0		
40	42	1	1	164.7	70.3	25.9	0	0	0	0	0	0	0	0	0	0	0	0	0	1	0	0	0	0	20130327	8.6	4	211828	0	0		
41	42	1	1				0	0	0	0	0	0	0	0	0	0	0	0	0	0	1	0	0	0	20140226	7.8	421	146156	0	0		
42	33	1	1				0	1	0	0	0	0	0	0	0	0	0	0	0	0	1	0	0	0	20140513	4.6	198	0	0	0		
43	66	1	1				0	1	1	0	0	0	0	0	0	0	0	0	0	1	0	0	0	0	20130422	7.8	62	18590	0	0		
44	23	1	1				0	1	0	0	0	0	0	0	0	0	0	0	0	0	1	0	0	0	20141215	6.3	454	17681	0	0		
45	65	0	1	160.9	53.9	20.8	0	0	0	0	0	0	0	0	0	0	0	0	0	0	0	1	0	0	20140916	4	275	70744	0	0		
46	45	1	1				0	1	0	0	0	0	0	0	0	0	0	0	0	0	0	1	0	0	20130708	5.6	121	0	0	0		

number	age	sex	nation	height	weight	BMI	LLV	underlygir	HTN	DM	CHD	MI	CVA	COPD	CLD	CKD	solid c	hematol	Charlson	I_INSTI	I_NNRTI	I_PI	I_BIC	I_DTG	ini_lab	ini_wbc	ini_CD4	ini_RNA	ini_HBV	ini_HCV	ini_NRTI_R	ini_INSTI_R
47	57	0	1				1	1	0	0	0	0	0	0	0	0	0	0	0	1	0	0	1	0	20191217	4.3	240	3383	0	0	0	0
48	34	1	1				1	1	0	1	0	0	0	1	0	0	0	0	2	1	0	0	0	0	20130913	4.2	494	4096	0	0		
49	47	1	1				0	1	0	0	0	0	0	0	0	0	0	0	0	1	0	0	0	0	20130827	4	12	1432268	0	0		
50	51	1	1				0	1	0	0	0	0	0	0	0	0	0	0	0	0	1	0	0	0	20130824	4.2	5	387193	0	0		
51	42	1	1				0	1	0	1	0	0	0	0	0	0	0	0	1	0	0	1	0	0	20130510	5.8	405	1500000	0	0		
52	56	1	1				0	1	0	0	0	0	0	0	1	0	0	0	1	0	0	1	0	0	20131023	4.5	75	0	0	1		
53	43	1	1				0	1	0	0	0	0	0	0	0	0	0	0	0	0	0	1	0	0	20131002	3.7	9	250000	0	0		
54	23	1	1				0	1	0	0	0	0	0	0	0	0	0	0	0	0	0	1	0	0	20131128	5.5	304	16287	0	0		
55	52	1	1				0	1	0	0	0	0	0	0	1	0	0	0	1	0	0	1	0	0	20140429	4.6	158	240	0	0		
56	23	1	1				0	0	0	0	0	0	0	0	0	0	0	0	0	0	0	1	0	0	20140527	5.9	145	58934	0	0		
57	52	1	1				0	1	1	0	0	0	0	0	0	0	0	0	0	0	0	1	0	0	20140513	5.7	70	0	0	0		
58	28	1	1				0	1	0	0	0	0	0	0	0	0	0	0	0	1	0	0	0	0	20151202	10.5	1031	3580	0	0	1	0
59	20	1	1				0	0	0	0	0	0	0	0	0	0	0	0	0	0	0	1	0	0	20140916	4.3	307	110406	0	0		
60	48	1	1				0	1	0	1	0	0	0	0	0	0	0	0	1	0	0	1	0	0	20140902	6.4	61	223379	0	0		
61	47	1	1				0	0	0	0	0	0	0	0	0	0	0	0	0	1	0	0	0	0	20140624	5.2	328	8603	0	0		
62	54	1	0	168	58.8	20.8	0	0	0	0	0	0	0	0	0	0	0	0	0	0	0	1	0	0	20140722	6.1	446	10527	0	0		
63	28	1	0				0	0	0	0	0	0	0	0	0	0	0	0	0	1	0	0	0	0	20140429	3.4	442	15379	0	0		
64	39	1	1				1	1	0	0	0	0	0	0	1	0	0	0	1	1	0	0	0	0	20140527	5.2	227	139084	0	0		
65	25	1	0				0	0	0	0	0	0	0	0	0	0	0	0	0	0	0	1	0	0	20140527	4.1	272	10406	0	0		
66	37	1	1				0	0	0	0	0	0	0	0	0	0	0	0	0	0	0	1	0	0	20140610	4.9	523	15587	0	0		
67	43	1	0				0	0	0	0	0	0	0	0	0	0	0	0	0	0	0	1	0	0	20140610	5.6	368	57564	0	0		
68	35	1	1	174	63	20.8	0	1	0	0	0	0	1	0	0	0	0	0	3	0	0	1	0	0	20140624	7.1	114	303	0	0		
69	36	1	0	174	69	22.8	0	1	0	0	0	0	0	0	0	0	0	0	0	1	0	0	0	0	20140708	3.8	155	103237	0	0		
70	33	1	1	176	48	15.5	0	0	0	0	0	0	0	0	0	0	0	0	0	0	0	1	0	0	20140722	8.7	21	57075	0	0		
71	19	1	1				0	1	0	0	0	0	0	0	0	0	0	0	0	1	0	0	0	0	20140805	8	98	1600000	0	0		
72	37	1	1				0	1	0	0	0	0	0	0	0	0	0	0	0	0	0	1	0	0	20140805	2.7	16	40000	0	0	0	0
73	46	1	1				0	0	0	0	0	0	0	0	0	0	0	0	0	0	0	1	0	0	20140805	6.3	375	11260	0	0		
74	69	1	1	170	46.7	16.2	0	1	1	0	0	0	0	0	0	0	0	0	0	1	0	0	0	0	20141107	4.8	499	257936	0	0		
75	52	1	1	171.7	55.2	18.7	0	1	0	0	0	0	0	0	0	0	0	0	1	1	0	0	0	0	20140902	10.7	605	36840	0	0		
76	25	1	1				0	0	0	0	0	0	0	0	0	0	0	0	0	1	0	0	0	0	20140902	5.6	260	5627	0	0		
77	29	1	1	171	51.8	17.7	0	0	0	0	0	0	0	0	0	0	0	0	0	1	0	0	0	0	20140929	2.7	105	386469	0	0		
78	56	1	1	166.4	54.2	19.6	0	0	0	0	0	0	0	0	0	0	0	0	0	0	0	1	0	0	20141014	4.8	95	28909	0	0		
79	36	1	0				0	0	0	0	0	0	0	0	0	0	0	0	0	1	0	0	0	0	20141212	4.9	336	8260	0	0		
80	30	1	0	171.7	62.5	21.2	0	0	0	0	0	0	0	0	0	0	0	0	0	0	0	1	0	0	20141028	5.1	286	0	0	0		
81	58	1	1	172	68	23.0	0	1	0	0	0	0	0	0	0	0	0	0	0	0	0	1	0	0	20141028	4.4	142	265	0	0		
82	55	1	1				0	0	0	0	0	0	0	0	0	0	0	0	0	1	0	0	0	0	20141125	6.4	376	718134	0	0		
83	18	1	1				0	1	0	0	0	0	0	0	0	0	0	0	0	1	0	0	0	0	20141111	5.2	368	146120	0	0		
84	25	1	1	181.2	58.5	17.8	0	1	0	0	0	0	0	0	0	0	0	0	0	1	0	0	0	0	20141125	7.1	504	33980	0	0		
85	50	1	1				0	0	0	0	0	0	0	0	0	0	0	0	0	0	0	1	0	0	20141223	3.1	90	915170	0	0		
86	24	1	1				0	0	0	0	0	0	0	0	0	0	0	0	0	1	0	0	1	0	20210319	4.1	335	14800	0	0	0	0
87	20	1	1				0	1	0	0	0	0	0	0	0	0	0	0	0	0	0	1	0	0	20150106	3.8	340	96500	0			
88	25	1	1				0	0	0	0	0	0	0	0	0	0	0	0	0	1	0	0	0	0	20160302	3	205	764	0	0		
89	42	1	0	183.3	82.6	24.6	0	0	0	0	0	0	0	0	0	0	0	0	0	1	0	0	0	0	20150120	8.3	250	233210	0	0		
90	36	1	0	166	56	20.3	0	0	0	0	0	0	0	0	0	0	0	0	0	1	0	0	0	0	20150120	4.2	15	582819	0	0		
91	31	1	0	173.4	81.2	27.0	0	0	0	0	0	0	0	0	0	0	0	0	0	1	0	0	0	0	20150203	5.8	315	1463	0	0		
92	25	1	0				0	1	0	0	0	0	0	0	0	0	0	0	0	1	0	0	0	0	20150203	5.1	327	13872	0	0		

number	age	sex	nation	height	weight	BMI	LLV	underlygir	HTN	DM	CHD	MI	CVA	COPD	CLD	CKD	solid c	hematolo	Charlson	I_INSTI	I_NNRTI	I_PI	I_BIC	I_DTG	ini_lab	ini_wbc	ini_CD4	ini_RNA	ini_HBV	ini_HCV	ini_NRTI_R	ini_INSTI_R
93	41	1	0	169.2	53.1	18.5	0	0	0	0	0	0	0	0	0	0	0	0	0	1	0	0	0	0	20150217	5.2	10	130857	0	0		
94	32	1	0				0	0	0	0	0	0	0	0	0	0	0	0	0	1	0	0	0	0	20150526	5.1	492	0				
95	20	1	1				0	0	0	0	0	0	0	0	0	0	0	0	0	1	0	0	0	0	20150331	5.6	238	66965	0	0		
96	31	0	0	155	58.2	24.2	0	0	0	0	0	0	0	0	0	0	0	0	0	1	0	0	0	0	20150827	4.2	449	7693	0	0		
97	30	1	0	162.2	75	28.5	0	0	0	0	0	0	0	0	0	0	0	0	0	1	0	0	0	0	20150729	3	366	13252	0	0		
98	50	1	1				0	1	0	0	0	0	0	0	0	0	0	0	0	1	0	0	0	0	20150526	5.4	373	109	0	0		
99	23	1	1				0	0	0	0	0	0	0	0	0	0	0	0	0	1	0	0	0	0	20170221	7	407	1090	0	0	1	0
100	25	1	1				0	0	0	0	0	0	0	0	0	0	0	0	0	1	0	0	0	0	20170919	5	408	1416	0	0	0	0
101	31	1	1				0	0	0	0	0	0	0	0	0	0	0	0	0	1	0	0	0	0	20151020	7.8	545	24850	0	0		
102	51	1	1	163.8	54.3	20.2	0	1	0	0	0	0	0	0	0	0	1	0	2	1	0	0	0	0	20150922	3.9	69	21000	1	0		
103	63	1	1	173	50	16.7	0	1	1	0	0	0	1	0	0	0	0	0	2	1	0	0	0	0	20150728	8.5	60	982977	0	0		
104	45	1	1				0	0	0	0	0	0	0	0	0	0	0	0	0	1	0	0	0	0	20150811	5.2	255	51126	0	0		
105	21	1	1				0	0	0	0	0	0	0	0	0	0	0	0	0	1	0	0	0	0	20150827	6.4	324	92985	0	0		
106	50	1	1	172	45.2	15.3	0	1	0	0	0	0	0	0	0	0	0	0	0	0	0	1	0	0	20151006	9	10	1103845	0	0	1	0
107	51	0	0	158.5	69.8	27.8	0	0	0	0	0	0	0	0	0	0	0	0	0	1	0	0	0	0	20151006	5.1	369	11310	0	0		
108	37	1	1	170	57.3	19.8	0	0	0	0	0	0	0	0	0	0	0	0	0	1	0	0	0	0	20151020	4.2	18	112896	0	0	1	0
109	47	1	1				0	1	0	1	0	0	0	0	1	0	0	0	2	1	0	0	0	0	20151020	5.5	549	1442	1	0		
110	26	0	0	163	53.2	20.0	0	1	0	0	0	0	0	0	0	0	0	0	0	1	0	0	0	0	20151103	5	497	23474	0	0		
111	51	1	1				0	1	0	0	0	0	0	0	1	0	0	0	1	1	0	0	0	0	20151201	6.2	991	1374	1	0	1	0
112	44	1	1				0	1	0	0	0	0	0	0	0	0	0	0	0	1	0	0	0	0	20151215	5.8	102	39897	0	0		
113	41	0	1				0	1	0	0	0	0	0	0	0	0	0	0	0	1	0	0	0	0	20151229	4.7	366	2352	0	0	0	0
114	75	1	1	160	53.5	20.9	0	1	1	1	0	1	0	0	0	0	0	1	4	1	0	0	0	0	20160112	6.3	82	3507	0	0	1	0
115	25	1	1				0	1	1	0	0	0	0	0	0	0	0	0	0	1	0	0	0	0	20160126	6.1	368	6704	0	0		
116	29	0	0	161.3	70.15	27.0	0	0	0	0	0	0	0	0	0	0	0	0	0	1	0	0	0	0	20160212	7.1	537	2198	0	0	0	0
117	16	1	1				0	0	0	0	0	0	0	0	0	0	0	0	0	1	0	0	0	0	20160212	4.3	379	3093	0	0	0	0
118	28	1	1				0	0	0	0	0	0	0	0	0	0	0	0	0	1	0	0	0	0	20160212	5.3	627	34013	0	0	0	0
119	30	0	0	163.5	81.3	30.4	0	0	0	0	0	0	0	0	0	0	0	0	0	1	0	0	0	0	20160308	2.9	212	96828	0	0	0	0
120	51	1	1	166.1	58.4	21.2	1	1	0	0	0	0	0	0	0	0	0	0	0	0	0	1	0	0	20110101	2.3	130	100	0	0	0	0
121	24	1	1				0	1	0	0	0	0	0	0	0	0	0	0	0	1	0	0	0	0	20160405	5.3	181	11229	0	0	0	0
122	37	1	1				0	0	0	0	0	0	0	0	0	0	0	0	0	1	0	0	0	0	20160405	5.8	623	61509	0	0	0	0
123	23	1	1	167.4	65	23.2	0	1	0	0	0	0	0	0	0	0	0	0	0	1	0	0	0	0	20160419	4.5	570	4797	0	0		
124	51	1	1	172.2	58.2	19.6	0	0	0	0	0	0	0	0	0	0	0	0	0	1	0	0	0	0	20160503	4.6	441	318	0	0		
125	41	1	1	172.3	71	23.9	0	1	0	0	0	0	0	0	1	0	0	0	1	1	0	0	1	0	20210210	9.5	25	569000	0	0	1	0
126	20	1	1				1	0	0	0	0	0	0	0	0	0	0	0	0	1	0	0	0	0	20151201	4.8	283	642000	0	0		
127	52	1	0				0	1	0	0	0	0	0	0	0	0	0	0	0	1	0	0	0	0	20160531	6.7	518	236	0	0		
128	41	1	1	173.5	56.6	18.8	0	0	0	0	0	0	0	0	0	0	0	0	0	1	0	0	0	0	20160614	8.1	59	324340	0	0		
129	32	1	1				0	0	0	0	0	0	0	0	0	0	0	0	0	1	0	0	0	0	20160726	3.6	236	87902	0	0		
130	39	0	1				0	1	1	0	0	0	0	0	0	0	0	0	1	1	0	0	0	0	20160809	2.5	56	559758	0	0		
131	42	1	1	174	74.75	24.7	0	0	0	0	0	0	0	0	0	0	0	0	0	1	0	0	0	0	20160920	3.5	82	377	0	0	0	0
132	29	1	1	166.9	54.9	19.7	0	0	0	0	0	0	0	0	0	0	0	0	0	1	0	0	0	0	20161101	9	476	125235	0	0		
133	39	1	1	173	73.7	24.6	0	1	0	0	0	0	0	0	0	0	0	0	0	1	0	0	0	0	20161115	21.5	1448	21070	0	0	0	0
134	51	1	1	160	35.6	13.9	0	0	0	0	0	0	0	0	0	0	0	0	0	1	0	0	0	0	20161129	7.4	18	93376	0	0	0	0
135	24	1	1				0	1	0	0	0	0	0	0	0	0	0	0	0	1	0	0	0	0	20161213	4.7	313	3192	0	0		
136	61	0	1	170	63	21.8	0	1	0	0	0	0	0	0	0	0	0	0	0	1	0	0	0	0	20161213	3.1	69	120	0	0		
137	28	1	0				0	0	0	0	0	0	0	0	0	0	0	0	0	1	0	0	0	0	20170110	2.5	91	68176	0	0		
138	41	1	1	178.6	72.9	22.9	0	0	0	0	0	0	0	0	0	0	0	0	0	1	0	0	0	0	20170124	3.7	153	3141280	0	0	0	0

number	age	sex	nation	height	weight	BMI	LLV	underlygir	HTN	DM	CHD	MI	CVA	COPD	CLD	CKD	solid c	hematolo	Charlson	I_INSTI	I_NNRTI	I_PI	I_BIC	I_DTG	ini_lab	ini_wbc	ini_CD4	ini_RNA	ini_HBV	ini_HCV	ini_NRTI_R	ini_INSTI_R
139	30	1	1	173	60	20.0	0	1	0	0	0	0	0	0	0	0	0	0	0	1	0	0	0	0	20170110	5.5	185	45454	0	0	0	0
140	43	0	0	158.5	63.7	25.4	0	0	0	0	0	0	0	0	0	0	0	0	0	1	0	0	0	0	20170207	4	155	87072	0	0		
141	41	0	0				0	0	0	0	0	0	0	0	0	0	0	0	0	1	0	0	0	0	20170221	7	555	3399	0	0		
142	24	0	0	164	85.3	31.7	0	0	0	0	0	0	0	0	0	0	0	0	0	1	0	0	0	0	20170221	6.3	904	50	0	0		
143	52	0	1	157	48	19.5	0	1	1	0	0	0	1	0	0	0	0	0	2	1	0	0	0	0	20170808	2.2	26	390954	0		0	0
144	31	1	1				0	1	0	0	0	0	0	0	0	0	0	0	0	1	0	0	0	0	20170307	6.2	459	6696	0	0		
145	26	1	1				0	0	0	0	0	0	0	0	0	0	0	0	0	1	0	0	0	0	20170307	3.8	350	9108	0	0		
146	50	1	1				0	0	0	0	0	0	0	0	0	0	0	0	0	1	0	0	0	0	20170321	1.8	16	964054	0	0		
147	30	1	0	165	64	23.5	0	0	0	0	0	0	0	0	0	0	0	0	0	1	0	0	0	0	20170404	7	666	1351	0	0	0	0
148	26	1	1	176	76	24.5	0	0	0	0	0	0	0	0	0	0	0	0	0	1	0	0	0	0	20170404	4.9	314	3148	0	0	0	0
149	65	1	1				0	1	1	0	0	0	0	0	0	0	0	0	0	1	0	0	1	0	20200214	3.1	90	204000	0	0	0	0
150	58	1	1				0	0	0	0	0	0	0	0	0	0	0	0	0	1	0	0	1	0	20211125	5.3	291	38300	0	0	0	1
151	21	1	1				0	0	0	0	0	0	0	0	0	0	0	0	0	1	0	0	0	0	20170530	5.8	297	158831	0	0	0	0
152	51	1	0	164	66.9	24.9	0	0	0	0	0	0	0	0	0	0	0	0	0	1	0	0	0	0	20170613	6.5	863	32499	0	0	0	0
153	21	1	1				0	1	0	0	0	0	0	0	0	0	0	0	0	1	0	0	0	0	20170613	4	372	50833	0	0	0	0
154	29	1	1	171.3	59.6	20.3	0	1	0	0	0	0	0	0	0	0	0	0	0	1	0	0	0	0	20170613	4.3	3	74141	0	0	0	0
155	26	1	1				0	0	0	0	0	0	0	0	0	0	0	0	0	1	0	0	0	0	20170627	2.5	24	499179	0	0	0	0
156	33	1	0				0	0	0	0	0	0	0	0	0	0	0	0	0	1	0	0	0	0	20171024	4	339	2700000	0	0		
157	29	1	1	174	78.7	26.0	0	0	0	0	0	0	0	0	0	0	0	0	0	1	0	0	0	0	20170725	3.6	48	1206447	0	0	0	0
158	26	1	1				0	0	0	0	0	0	0	0	0	0	0	0	0	1	0	0	0	0	20170808	9	567	35359	0	0	0	0
159	30	0	0	160	66	25.8	0	0	0	0	0	0	0	0	0	0	0	0	0	1	0	0	0	0	20170808	4.2	240	2002	1	0	0	0
160	32	1	1				1	0	0	0	0	0	0	0	0	0	0	0	0	1	0	0	0	0	20170905	5.5	364	1060461	0	0		
161	27	0	0	155.8	55.5	22.9	0	0	0	0	0	0	0	0	0	0	0	0	0	1	0	0	0	0	20170905	4.9	819	1094	0	0	0	0
162	33	1	1				0	1	0	0	0	0	0	0	0	0	0	0	0	1	0	0	0	0	20170905	5.3	426	189095	0	0	0	0
163	27	1	1				0	0	0	0	0	0	0	0	0	0	0	0	0	1	0	0	0	0	20171010	5.4	862	6160	0	0	0	0
164	35	0	0	155	60	25.0	0	0	0	0	0	0	0	0	0	0	0	0	0	1	0	0	0	0	20171025	2.9	89	289821	0	0	0	0
165	24	1	1				0	0	0	0	0	0	0	0	0	0	0	0	0	1	0	0	0	0	20171024	4	364	9275	0	0	0	0
166	21	1	0	173.2	68.6	22.9	0	0	0	0	0	0	0	0	0	0	0	0	0	1	0	0	0	0	20171108	3.7	208	88980	0	0	0	0
167	47	1	1				0	0	0	0	0	0	0	0	0	0	0	0	0	1	0	0	0	0	20171108	6.1	372	40586	0	0	0	0
168	39	1	1				0	0	0	0	0	0	0	0	0	0	0	0	0	1	0	0	0	0	20171205	4.1	130	107656	0	0	0	0
169	42	1	1	172	67	22.6	0	0	0	0	0	0	0	0	0	0	0	0	0	1	0	0	0	0	20171219	2.4	24	29814	0	0		
170	45	1	0	167.8	77.6	27.6	0	1	0	0	0	0	0	0	1	0	0	0	1	1	0	0	0	0	20180116	3.3	189	2816	0	1		
171	21	1	0				0	0	0	0	0	0	0	0	0	0	0	0	0	1	0	0	0	0	20180130	3.7	423	11401	0	0	0	0
172	60	1	1	170	58.2	20.1	0	0	0	0	0	0	0	0	0	0	0	0	0	1	0	0	0	0	20180213	10.6	16	8654	0	0	0	0
173	34	1	1				0	1	0	0	0	0	0	0	1	0	0	0	1	1	0	0	0	0	20180227	5.1	241	6979	0	0	1	0
174	29	1	1				0	1	0	0	0	0	0	0	0	0	0	0	0	1	0	0	0	0	20180313	4.1	350	14425	0	0	0	0
175	27	1	1				0	0	0	0	0	0	0	0	0	0	0	0	0	1	0	0	0	0	20180313	6	637	3563	0	0	0	0
176	32	0	0	156.1	67.5	27.7	0	1	0	0	0	0	0	0	0	0	0	0	0	1	0	0	0	0	20180327	2.6	304	1807	1	0		
177	42	1	1	172	58	19.6	0	1	0	0	0	0	0	0	0	0	0	0	0	1	0	0	0	0	20180424	2.9	15	121429	0	0		
178	39	0	1				0	1	0	0	0	0	0	0	0	0	0	0	0	1	0	0	0	0	20180508	5.3	49	898000	0	0		
179	32	1	1				0	1	1	1	0	0	0	0	0	0	1	0	3	1	0	0	0	0	20180605	2.9	181	792733	0	0	0	0
180	55	1	0	176	52	16.8	0	1	0	0	0	0	0	0	0	0	0	0	0	1	0	0	0	0	20180605	3.7	52	46776	0	0	0	0
181	53	0	0				0	1	0	0	0	0	0	0	1	0	0	0	0	1	0	0	0	0	20180619	6	661	337	1	0		
182	24	1	1				0	1	0	0	0	0	0	0	0	0	0	0	0	1	0	0	0	0	20180619	4.1	275	53868	0	0	0	0
183	34	1	1				0	0	0	0	0	0	0	0	0	0	0	0	0	1	0	0	0	0	20180703	5.6	287	74883	0	0	0	0
184	39	1	1				0	1	0	0	0	0	0	0	0	0	0	0	0	1	0	0	0	0	20180731	3.7	51	42006	0	0	0	0

number	age	sex	nation	height	weight	BMI	LLV	underlygir	HTN	DM	CHD	MI	CVA	COPD	CLD	CKD	solid c	hematolc	Charlson	I_INSTI	I_NNRTI	I_PI	I_BIC	I_DTG	ini_lab	ini_wbc	ini_CD4	ini_RNA	ini_HBV	ini_HCV	ini_NRTI_R	ini_INSTI_R
185	24	1	1				0	0	0	0	0	0	0	0	0	0	0	0	0	1	0	0	0	0	20180814	4.4	466	11555	0	0	0	0
186	53	1	0	167.6	86.6	30.8	1	1	1	0	0	0	0	0	0	0	0	0	0	1	0	0	0	0	20180814	3.3	107	11790	0	0	0	0
187	28	1	0	166.9	59.4	21.3	0	0	0	0	0	0	0	0	0	0	0	0	0	1	0	0	0	0	20180911	6.2	490	3132	0	0	0	0
188	32	1	1	169.1	55	19.2	0	0	0	0	0	0	0	0	0	0	0	0	0	1	0	0	0	0	20180921	8	361	332	0	0		
189	34	0	0	156.8	72.6	29.5	0	0	0	0	0	0	0	0	0	0	0	0	0	1	0	0	0	0	20180921	4.9	481	0	0	0		
190	56	1	1	153	48	20.5	0	1	1	1	0	0	0	0	0	0	0	0	3	1	0	0	0	0	20181106	7.2	395	84803	0	0	0	0
191	32	1	1				0	0	0	0	0	0	0	0	0	0	0	0	0	1	0	0	0	0	20181008	6.7	559	22516	0	0	0	0
192	35	0	0	156.6	100.5	41.0	0	0	0	0	0	0	0	0	0	0	0	0	0	1	0	0	0	0	20181023	4.8	328	0	0	0		
193	31	1	1				0	1	0	0	0	0	0	0	0	0	0	0	0	1	0	0	0	0	20181106	4.9	224	48943	0	0	0	0
194	47	1	0	183.7	81.7	24.2	0	0	0	0	0	0	0	0	0	0	0	0	0	1	0	0	0	0	20181106	3.6	522	3155	0	0	0	1
195	56	1	1				0	0	0	0	0	0	0	0	0	0	0	0	0	1	0	0	0	0	20181121	6.3	215	821	0	0		
196	38	1	1				0	1	1	0	0	0	0	0	0	0	0	0	0	1	0	0	0	0	20181231	3.8	354	1319	0	0		
197	29	1	1	180.7	56.2	17.2	0	0	0	0	0	0	0	0	0	0	0	0	0	1	0	0	0	0	20181231	5.4	39	90835	0	0	0	0
198	37	1	0	173.8	70	23.2	0	0	0	0	0	0	0	0	0	0	0	0	0	1	0	0	0	0	20181231	5.9	9	84400	0	0		
199	25	1	1				0	0	0	0	0	0	0	0	0	0	0	0	0	1	0	0	0	0	20190115	7.3	464	9091	0	0		
200	37	1	0	185	80.1	23.4	0	0	0	0	0	0	0	0	0	0	0	0	0	1	0	0	0	0	20190115	5.5	443	46645	0	0	0	0
201	21	1	1				0	0	0	0	0	0	0	0	0	0	0	0	0	1	0	0	0	0	20190115	5.4	422	24961	0	0	0	0
202	40	0	0	166	80	29.0	0	1	0	0	0	0	0	0	0	0	0	0	0	1	0	0	0	0	20190129	4.2	933	163	0	0		
203	29	1	0	173	68.5	22.9	1	0	0	0	0	0	0	0	0	0	0	0	0	1	0	0	0	0	20190129	7.3	366	84223	0	0	0	0
204	24	1	1				0	0	0	0	0	0	0	0	0	0	0	0	0	1	0	0	0	0	20190129	6.5	639	636	0	0	0	0
205	44	0	1				0	1	0	0	0	0	0	0	0	0	0	0	0	1	0	0	0	0	20190226	3.1	126	90835	0	0	0	0
206	25	1	0				1	0	0	0	0	0	0	0	0	0	0	0	0	1	0	0	0	0	20190402	6.2	189	8905	0	0	0	0
207	41	1	0	187	89.5	25.6	0	1	1	0	0	0	0	0	0	0	0	0	0	1	0	0	0	0	20190402	3.2	376	2603	0	0	0	0
208	31	1	1				0	0	0	0	0	0	0	0	0	0	0	0	0	1	0	0	0	0	20190326	6	294	97296	0	0	0	0
209	40	0	0	159.5	111.9	44.0	0	0	0	0	0	0	0	0	0	0	0	0	0	1	0	0	0	0	20190326	5.9	602	615	0	0	0	0
210	28	1	0	178	72.3	22.8	0	0	0	0	0	0	0	0	0	0	0	0	0	1	0	0	0	0	20190423	3.6	417	23699	0	0	0	1
211	35	1	1				0	0	0	0	0	0	0	0	0	0	0	0	0	1	0	0	0	0	20190423	5.3	32	194448	0	0	0	0
212	24	0	0	157	61	24.7	0	0	0	0	0	0	0	0	0	0	0	0	0	1	0	0	0	0	20190604	7	98	125206	0	0	0	0
213	32	1	0				0	0	0	0	0	0	0	0	0	0	0	0	0	1	0	0	0	0	20190618	5	3	434809	0	0	1	0
214	25	1	1				0	1	0	0	0	0	0	0	0	0	0	0	0	1	0	0	0	0	20190618	11.7	21	604930	0	0		
215	31	0	0	169.2	83.9	29.3	0	0	0	0	0	0	0	0	0	0	0	0	0	0	0	1	0	0	20190702	5.9	764	6399	0	0	0	1
216	27	1	1				0	0	0	0	0	0	0	0	0	0	0	0	0	1	0	0	0	0	20191119	6.7	647	849	0	0	1	0
217	25	1	1				0	0	0	0	0	0	0	0	0	0	0	0	0	1	0	0	0	0	20190730	4.4	352	7119	0	0	0	0
218	27	1	1				0	0	0	0	0	0	0	0	0	0	0	0	0	1	0	0	0	0	20190813	4.1	390	1370	0	0		
219	32	1	0	163.7	67	25.0	0	0	0	0	0	0	0	0	0	0	0	0	0	1	0	0	0	0	20190813	6.2	253	4659	0	0	0	0
220	25	1	1				0	1	0	0	0	0	0	0	0	0	0	0	0	1	0	0	0	0	20190924	5	264	8740	0	0		
221	38	0	0	163.8	77.1	28.7	0	0	0	0	0	0	0	0	0	0	0	0	0	1	0	0	0	0	20191008	6	772	0	0	0		
222	36	1	1				0	0	0	0	0	0	0	0	0	0	0	0	0	1	0	0	0	0	20191008	3.6	113	90592	0	0	0	0
223	38	1	0	188	89.2	25.2	0	0	0	0	0	0	0	0	0	0	0	0	0	1	0	0	0	0	20191022	4.5	64	24044	0	0	1	1
224	39	1	0	168.1	75.9	26.9	0	1	1	1	0	0	0	0	0	0	0	0	1	1	0	0	0	0	20191022	5.1	241	55751	0	0	0	0
225	29	1	1	167.9	62.3	22.1	1	0	0	0	0	0	0	0	0	0	0	0	0	1	0	0	1	0	20191022	5.9	59	106748	0	0	0	0
226	27	1	1				0	1	0	0	0	0	0	0	0	0	0	0	0	1	0	0	1	0	20191022	8.3	428	3643	0	0	1	0
227	50	1	1	175	48	15.7	0	0	0	0	0	0	0	0	0	0	0	0	0	1	0	0	1	0	20191022	11.8	61	19,955,072	0	0		
228	28	0	0	172.6	91.2	30.6	0	0	0	0	0	0	0	0	0	0	0	0	0	1	0	0	1	0	20191217	6.7	602	3874	0	0		
229	23	1	0	180.3	85	26.1	0	0	0	0	0	0	0	0	0	0	0	0	0	1	0	0	1	0	20191217	7.2	322	37284	0	0	0	0
230	49	1	0	171.8	73.3	24.8	0	0	0	0	0	0	0	0	0	0	0	0	0	1	0	0	1	0	20191217	3.4	222	1940	0	0	0	0

number	age	sex	nation	height	weight	BMI	LLV	underlygir	HTN	DM	CHD	MI	CVA	COPD	CLD	CKD	solid c	hematolc	Charlson	I_INSTI	I_NNRTI	I_PI	I_BIC	I_DTG	ini_lab	ini_wbc	ini_CD4	ini_RNA	ini_HBV	ini_HCV	ini_NRTI_R	ini_INSTI_R
231	39	1	0	174.5	70.6	23.2	0	0	0	0	0	0	0	0	0	0	0	0	0	1	0	0	1	0	20191203	9.5	730	449	0	0		
232	21	1	0				0	0	0	0	0	0	0	0	0	0	0	0	0	1	0	0	1	0	20191217	4.4	216	75971	0	0		
233	29	1	1				0	0	0	0	0	0	0	0	0	0	0	0	0	1	0	0	1	0	20200114	4.3	526	25163	0	0	0	0
234	27	1	1				0	0	0	0	0	0	0	0	0	0	0	0	0	1	0	0	1	0	20200128	7.1	686	4260	0	0		
235	60	1	1	161.9	49.6	18.9	0	1	1	0	0	0	0	0	1	0	0	0	1	1	0	0	0	0	20200205	4.6	249	71475	0	0	0	0
236	29	1	1				0	1	0	0	0	0	0	0	0	0	0	0	0	1	0	0	1	0	20210729	6.1	468	98200	0	0	0	1
237	28	1	1	173	67	22.4	0	0	0	0	0	0	0	0	0	0	0	0	0	1	0	0	1	0	20200514	6.4	462	1930	0	0		
238	23	1	1				0	0	0	0	0	0	0	0	0	0	0	0	0	1	0	0	1	0	20200529	7.7	654	10200	0	0	0	0
239	40	1	1				0	0	0	0	0	0	0	0	0	0	0	0	0	1	0	0	1	0	20200611	4.3	312	12200	0	0	0	0
240	30	1	1				0	1	0	0	0	0	0	0	0	0	0	0	0	1	0	0	1	0	20200902	5.1	176	194000	0	0		
241	41	0	0	162.2	56.3	21.4	0	0	0	0	0	0	0	0	0	0	0	0	0	1	0	0	1	0	20200820	3.2	345	40500	0	0		
242	18	1	1				0	0	0	0	0	0	0	0	0	0	0	0	0	1	0	0	1	0	20200902	6.4	684	124	0	0		
243	43	1	0	172	72	24.3	0	0	0	0	0	0	0	0	0	0	0	0	0	1	0	0	1	0	20200916	4	104	183000	0	0		
244	32	1	0	180	84	25.9	0	1	0	1	0	0	0	0	0	0	0	0	1	1	0	0	0	0	20200929	4.5	70	573	0	0		
245	80	1	1	163	52	19.6	1	1	0	1	0	0	0	0	0	0	0	0	2	1	0	0	1	0	20201118	4.5	127	682000	0	0	0	1
246	42	1	1	161.3	61	23.4	1	0	0	0	0	0	0	0	0	0	0	0	0	1	0	0	1	0	20201030	5.3	29	1140000	0	0	0	0
247	35	1	1				0	1	0	0	0	0	0	0	0	0	0	0	0	1	0	0	1	0	20201203	2.5	124	81000	0	0	0	0
248	30	1	1				0	0	0	0	0	0	0	0	0	0	0	0	0	1	0	0	1	0	20210114	4.6	289	9790	0	0	0	0
249	39	1	0	163.3	67.8	25.4	0	0	0	0	0	0	0	0	0	0	0	0	0	1	0	0	1	0	20210114	6.4	595	291000	0	0	1	0
250	40	0	0	152.2	68.02	29.4	0	0	0	0	0	0	0	0	0	0	0	0	0	1	0	0	1	0	20210128	5.5	525	6840	0	0	1	0
251	45	1	0	176.7	86.2	27.6	0	1	0	0	0	0	0	0	0	1	0	0	2	1	0	0	1	0	20210128	5.1	492	7540	0	1	1	0
252	41	1	0	166.7	73.1	26.3	0	1	0	1	0	0	0	0	0	0	0	0	1	1	0	0	1	0	20210218	8	454	269000	1	0	0	0
253	46	1	0	158.7	63.1	25.1	0	1	0	0	0	0	0	0	0	0	0	0	1	1	0	0	1	0	20201125	6.9	61	4408	0	0		
254	54	1	0	173.7	67.3	22.3	0	0	0	0	0	0	0	0	0	0	0	0	0	1	0	0	1	0	20210319	5.2	347	161000	1	1	0	0
255	35	1	1	172	65.7	22.2	0	0	0	0	0	0	0	0	0	0	0	0	0	1	0	0	1	0	20210401	3.4	159	177000	0	0	0	0
256	43	1	0	172	70	23.7	0	0	0	0	0	0	0	0	0	0	0	0	0	1	0	0	0	1	20210401	5.4	285	2840	0	0	0	0
257	34	1	0	162	68.2	26.0	0	0	0	0	0	0	0	0	0	0	0	0	0	1	0	0	1	0	20171031	10.6	60	149120	0	0	0	0
258	34	1	0				0	0	0	0	0	0	0	0	0	0	0	0	0	1	0	0	1	0	20210415	3.3	553	1250	0	0		
259	27	1	1				0	0	0	0	0	0	0	0	0	0	0	0	0	1	0	0	0	0	20210507	7.5	322	21500	0	0	0	0
260	27	1	1				0	1	0	0	0	0	0	0	0	0	0	0	0	1	0	0	0	1	20200401	4.1	381	18700	0	0	0	0
261	31	1	0	158.2	55	22.0	0	1	0	0	0	0	0	0	0	0	0	0	0	1	0	0	1	0	20210503	7.6	365	16100	0	0		
262	29	1	0	176.2	66.8	21.5	0	0	0	0	0	0	0	0	0	0	0	0	0	1	0	0	1	0	20210510	4.4	480	290633	0	0		
263	42	0	0				0	1	0	0	0	0	0	0	0	0	0	1	0	1	0	0	0	0	20210514	4.2	106	81	0	0		
264	31	0	0	156	53	21.8	0	1	0	0	0	0	0	0	0	0	0	0	0	1	0	0	0	0	20210531	3.1	40	500000	0	0		
265	29	0	0	155	85	35.4	0	0	0	0	0	0	0	0	0	0	0	0	0	1	0	0	1	0	20210617	4.8	503	1470	0	0		
266	31	1	0	162	65.9	25.1	0	0	0	0	0	0	0	0	0	0	0	0	0	1	0	0	1	0	20210701	4.1	93	162000	0	0	0	0
267	57	1	1	167	71.1	25.5	0	0	0	0	0	0	0	0	0	0	0	0	2	1	0	0	1	0	20210716	3.3	34	329	0	0		
268	30	1	1				0	0	0	0	0	0	0	0	0	0	0	0	0	1	0	0	1	0	20211230	4.9	692	43800	0	0	0	0
269	45	1	1	167	53.2	19.1	0	0	0	0	0	0	0	0	0	0	0	0	0	1	0	0	1	0	20210805	4.8	1	123000	0	0	0	0
270	57	1	1				0	0	0	0	0	0	0	0	0	0	0	0	0	1	0	0	1	0	20210812	4.6	258	19700	0	0	0	1
271	49	1	1				0	1	0	0	0	0	0	0	0	0	0	0	0	1	0	0	0	1	20210827	2	20	44800	0	0		
272	27	1	1				0	0	0	0	0	0	0	0	0	0	0	0	0	1	0	0	1	0	20210826	7.5	524	370000	0	0	0	0
273	31	1	0	170	64	22.1	0	1	0	0	0	0	0	0	0	0	0	0	0	1	0	0	1	0	20211007	8.7	262	42300	0	0		
274	32	1	1				0	0	0	0	0	0	0	0	0	0	0	0	0	1	0	0	0	0	20211209	5.4	166	35836	0	0		
275	27	1	1				0	0	0	0	0	0	0	0	0	0	0	0	0	1	0	0	1	0	20211230	3.8	324	14800	0	0	0	0
276	27	1	1				0	1	0	0	0	0	0	0	0	0	0	0	0	1	0	0	1	0	20220120	4.9	206	581000	0	0	0	0

number	age	sex	nation	height	weight	BMI	LLV	underlygir	HTN	DM	CHD	MI	CVA	COPD	CLD	CKD	solid c	hematol	Charlson	I_INSTI	I_NNRTI	I_PI	I_BIC	I_DTG	ini_lab	ini_wbc	ini_CD4	ini_RNA	ini_HBV	ini_HCV	ini_NRTI_R	ini_INSTI_R
277	33	1	0	185	77	22.5	0	0	0	0	0	0	0	0	0	0	0	0	0	1	0	0	1	0	20220203	4.9	273	23100	0	0	0	0
278	20	1	0	158	55	22.0	0	0	0	0	0	0	0	0	0	0	0	0	0	1	0	0	0	0	20220317	4.7	3	1210000	0	1	0	0
279	26	1	0	151	47	20.6	0	0	0	0	0	0	0	0	0	0	0	0	0	1	0	0	0	1	20220428	11.1	285	25200	0	0	0	0
280	26	1	1				0	0	0	0	0	0	0	0	0	0	0	0	0	1	0	0	0	0	20220421	8.2	538	21800	0	0	0	0
281	53	1	1	179	78	24.3	0	1	1	0	0	0	0	0	0	0	0	0	0	1	0	0	1	0	20220512	6.3	20	4320000	0	0	0	0
282	29	0	0	154.7	55.8	23.3	0	1	0	0	0	0	0	0	1	0	0	0	1	1	0	0	0	0	20220330	6.2	431	2375	0	0	0	0
283	32	1	0	166.3	59.8	21.6	0	1	0	0	0	0	0	0	1	0	0	0	1	1	0	0	0	1	20220616	3.8	1015	0	0	0		
284	34	1	1				0	1	0	1	0	0	0	0	0	0	0	0	1	1	0	0	1	0	20220623	8.5	806	28400	0	0	0	0
285	24	1	1				0	0	0	0	0	0	0	0	0	0	0	0	0	1	0	0	0	0	20161031	10	187	176000	0	0	0	0
286	51	1	1				0	0	0	0	0	0	0	0	0	0	0	0	1	0	1	0	0	0	20160211	9	67	113000	1	0		
287	25	1	1				0	0	0	0	0	0	0	0	0	0	0	0	0	1	0	0	1	0	20210823	7.2	553	3820	0	0		
288	38	1	1				0	1	0	1	0	0	0	0	0	0	0	0	1	1	0	0	1	0	20221213	9.1	275	210,201	0	0	0	0
289	29	1	1				0	0	0	0	0	0	0	0	0	0	0	0	0	1	0	0	1	0	20200626	5.2	289	953000	0	0	0	0
290	66	1	1				0	1	0	1	0	0	0	0	0	0	0	0	3	1	0	0	0	0	20180914	4.2	165	94,000	0	0		
291	56	1	1				0	1	0	1	0	0	1	0	0	0	0	0	3	0	0	1	0	0	20090907	9.6	246	15200	0	0		
292	63	1	1				0	1	1	0	0	1	0	0	0	0	0	0	3	0	0	1	0	0	20150317	6.3	37	7300000	0	0		
293	43	1	1				0	1	1	0	0	0	0	0	0	0	0	0	0	1	0	0	0	0	20150813	6.9	27	398,000	0			
294	22	1	1				0	0	0	0	0	0	0	0	0	0	0	0	0	1	0	0	0	0	20190805	4.3	266	217,000	0	0	0	0
295	31	1	1				0	0	0	0	0	0	0	0	0	0	0	0	0	1	0	0	0	0	20141111	3.8	286	14600	0	0		
296	22	1	1				0	0	0	0	0	0	0	0	0	0	0	0	0	1	0	0	0	0	20190325	4.7	272	2610	0	0	0	0
297	34	1	1				0	0	0	0	0	0	0	0	0	0	0	0	0	1	0	0	1	0	20210104	4.2	297	68300	1	0		
298	68	1	1				0	1	0	1	0	0	0	0	0	0	0	0	3	1	0	0	1	0	20230227	9.3	94	62,177	0	0	0	0
299	20	1	1				0	0	0	0	0	0	0	0	0	0	0	0	0	0	0	1	0	0	20150608	4	618	0				
300	48	0	1				0	1	1	1	0	0	0	0	1	0	1	0	4	1	0	0	0	0	20110816	6	260	73600	0	0		
301	33	1	1				0	0	0	0	0	0	0	0	0	0	0	0	0	1	0	0	0	0	20200207	9.5	244	228000	0	0		
302	58	1	1				0	0	0	0	0	0	0	0	0	0	0	0	1	1	0	0	0	0	20161109	7	451	2,300,000	0	0		
303	20	1	1				0	0	0	0	0	0	0	0	0	0	0	0	0	0	0	1	0	0	20090720	7.6	295	3460	0	0		
304	32	0	1				0	0	0	0	0	0	0	0	0	0	0	0	0	0	0	1	0	0	20130607	7.9	322	1220	1	0		
305	23	1	1				0	0	0	0	0	0	0	0	0	0	0	0	0	1	0	0	1	0	20221216	4	326	7990	0	0	0	1
306	30	1	1				0	0	0	0	0	0	0	0	0	0	0	0	0	1	0	0	1	0	20200811	6.8	649	324	0	0	0	0
307	19	1	1				0	0	0	0	0	0	0	0	0	0	0	0	0	0	0	1	0	0	20130225	6.6	312	28200	0	1		
308	26	1	1				0	0	0	0	0	0	0	0	0	0	0	0	0	1	0	0	1	0	20210923	4.8	427	3,060,000	0	0		
309	28	1	1				0	0	0	0	0	0	0	0	0	0	0	0	0	1	0	0	0	0	20141014	4.7	64	299000	0	0		
310	18	1	1				0	0	0	0	0	0	0	0	0	0	0	0	0	0	0	1	0	0	20131108	6.6	137	26300	0	0		
311	21	1	1				0	0	0	0	0	0	0	0	0	0	0	0	0	0	0	1	0	0	20110608	5	228	89400	0	0		
312	29	1	1				0	0	0	0	0	0	0	0	0	0	0	0	0	0	0	1	0	0	20090521	7.7	227	11756	0	0		
313	66	1	1				0	0	0	0	0	0	0	0	0	0	0	0	0	0	0	1	0	0	20060315	6	20	29000	0	0		
314	19	1	1				0	0	0	0	0	0	0	0	0	0	0	0	0	0	0	1	0	0	20130717	9.7	208	10200	0	0		
315	37	1	1				0	0	0	0	0	0	0	0	0	0	0	0	0	0	0	1	0	0	20120725	6.8	115	518000	0	0		
316	57	1	1				0	1	1	1	0	1	0	0	0	0	0	0	3	0	0	1	0	0	20170316	6.8	6	119,000	0	0		
317	33	1	1				0	0	0	0	0	0	0	0	0	0	0	0	0	0	0	1	0	0	20150112	4.9	75	57,000	0	0		
318	43	1	1				0	0	0	0	0	0	0	0	0	0	0	0	0	0	0	1	0	0	20080123	7.6	80	90000	0	0		
319	41	1	1				0	0	0	0	0	0	0	0	0	0	0	0	0	0	0	1	0	0	20170404	13.7	29	235000	0	0		
320	46	1	1				0	0	0	0	0	0	0	0	0	0	0	0	0	0	0	1	0	0	20090109	6.4	212	13594	0	0		
321	39	1	1				0	0	0	0	0	0	0	0	0	0	0	0	0	0	0	1	0	0	20090825	6.5	160	79700	0	0		
322	50	1	1				0	1	0	0	0	0	0	0	0	0	1	0	3	0	0	1	0	0	20090825	5.3	190	72000	0	0		

number	age	sex	nation	height	weight	BMI	LLV	underlygir	HTN	DM	CHD	MI	CVA	COPD	CLD	CKD	solid c	hematolo	Charlson	I_INSTI	I_NNRTI	I_PI	I_BIC	I_DTG	ini_lab	ini_wbc	ini_CD4	ini_RNA	ini_HBV	ini_HCV	ini_NRTI_R	ini_INSTI_R
323	81	1	1				0	1	0	1	0	0	0	0	0	0	0	0	5	0	0	1	0	0	20200128	5.4	12	226000	0	0		
324	27	1	1				0	0	0	0	0	0	0	0	0	0	0	0	0	1	0	0	0	1	20211123	7.5	312	365,738	0	0	0	0
325	34	1	1				0	0	0	0	0	0	0	0	0	0	0	0	0	0	0	1	0	0	20110928	8.1	198	20400	0	0		
326	24	1	1				0	0	0	0	0	0	0	0	0	0	0	0	0	0	0	1	0	0	20180319	3.1	120	21,900	0	0	0	0
327	48	1	1				0	0	0	0	0	0	0	0	0	0	0	0	0	0	0	1	0	0	20101229	9.6	271	69100	0	0		
328	45	1	1				0	1	0	1	0	0	0	0	0	0	0	0	1	0	1	0	0	0	20110222	5.8	270	91000	0	0		
329	69	1	1				0	1	1	0	0	0	0	0	0	0	0	0	2	0	0	1	0	0	20110511	5.2	91	1150000	0	0		
330	33	1	1				0	0	0	0	0	0	0	0	0	0	0	0	0	0	0	1	0	0	20110906	9	265	101000	0	0		
331	48	1	1				0	0	0	0	0	0	0	0	0	0	0	0	0	0	0	1	0	0	20111006	6.8	185	6990	0	0		
332	62	1	1				0	1	1	1	0	0	0	0	0	1	0	0	3	1	0	0	0	0	20111117	7.3	222	47700	0	0		
333	59	1	1				0	0	0	0	0	0	0	0	0	0	0	0	1	0	0	1	0	0	20120625	5.3	47	543000	0	1		
334	27	1	1				0	0	0	0	0	0	0	0	0	0	0	0	0	0	1	0	0	0	20130716	11.9	336	38200	0	0		
335	49	1	1				0	0	0	0	0	0	0	0	0	0	0	0	0	0	1	0	0	0	20141001	7	338	10000000	1	0		
336	30	1	0				0	0	0	0	0	0	0	0	0	0	0	0	0	1	0	0	1	0	20200924	3.9	180	128000	0	0	0	0
337	26	1	1				0	0	0	0	0	0	0	0	0	0	0	0	0	1	0	0	0	0	20130302	4.5	21	101000	0	0		
338	42	1	1				0	0	0	0	0	0	0	0	0	0	0	0	0	0	1	0	0	0	20140520	4.5	422	57500	0	0		
339	42	1	1				0	1	0	0	0	0	1	0	0	0	0	0	1	1	0	0	0	0	20130731	5.9	28	162000	0	0		
340	74	1	1				0	1	1	0	0	0	0	0	0	1	0	0	3	1	0	0	0	0	20170529	5	312	193,000	0	0		
341	31	1	1				0	1	0	0	0	0	0	0	0	1	0	0	0	0	0	1	0	0	20130424	7.2	25	109000	0	0		
342	37	0	1				0	0	0	0	0	0	0	0	0	0	0	0	0	0	1	0	0	0	20130624	6	62	353000	0	0		
343	30	1	1				0	0	0	0	0	0	0	0	0	0	0	0	0	0	1	0	0	0	20131109	7.3	124	264000	0	0		
344	43	1	1				0	0	0	0	0	0	0	0	0	0	0	0	0	1	0	0	0	0	20190123	2.3	4	114,000	0	0	0	0
345	40	1	1				0	0	0	0	0	0	0	0	0	0	0	0	0	0	0	0	0	0	20180213	6.8	184	21200	0	0		
346	21	1	1				0	0	0	0	0	0	0	0	0	0	0	0	0	0	1	0	0	0	20131219	7.1	262	25800	0	0		
347	26	1	1				0	0	0	0	0	0	0	0	0	0	0	0	0	0	0	1	0	0	20140205	5.3	111	125000	0	0		
348	22	1	1				0	0	0	0	0	0	0	0	0	0	0	0	0	0	0	1	0	0	20150422	3	147	1170000	0	0		
349	34	1	1				0	0	0	0	0	0	0	0	0	0	0	0	0	1	0	0	1	0	20200909	6.5	724	7650	0	0	0	0
350	34	0	1				0	0	0	0	0	0	0	0	0	0	0	0	0	0	1	0	0	0	20140705	7.6	346	27700	0			
351	38	1	1				0	0	0	0	0	0	0	0	0	0	0	0	0	0	0	0	0	0	20150521	10.6	1128	0	0			
352	52	1	1				1	1	0	0	0	0	0	0	0	0	0	1	3	0	0	1	0	0	20140804	5	87	67000	0	0		
353	26	1	1				0	0	0	0	0	0	0	0	0	0	0	0	0	0	0	1	0	0	20150122	11.8	136	104000	0	0		
354	52	1	1				0	1	1	1	0	0	0	0	0	0	0	0	2	0	1	0	0	0	20150517	6.7	19	95200	0	0	0	0
355	44	1	1				0	0	0	0	0	0	0	0	0	0	0	0	0	0	0	1	0	0	20150701	2.7	33	229,000	0	0		
356	23	1	1				0	0	0	0	0	0	0	0	0	0	0	0	0	0	1	0	0	0	20150924	4.3	129	88,900	0	0	0	0
357	55	1	1				0	0	0	0	0	0	0	0	0	0	0	0	1	1	0	0	1	0	20221025	6.3	127	120,238	0	0		
358	60	1	1				0	0	0	0	0	0	0	0	0	0	0	0	2	0	1	0	0	0	20161031	4.9	40	142000	0	0		
359	36	1	0				0	0	0	0	0	0	0	0	0	0	0	0	0	0	1	0	0	0	20170116	8.3	603	65,500	0	0		
360	31	1	1				1	0	0	0	0	0	0	0	0	0	0	0	0	1	0	0	0	0	20191029	5.1	136	1150000	0	0	0	1
361	51	1	1				0	0	0	0	0	0	0	0	0	0	0	0	1	0	1	0	0	0	20180118	7.4	881	36208	0			
362	67	1	1				0	0	0	0	0	0	0	0	0	0	0	0	2	1	0	0	0	0	20180903	3.8	81	72300	0	0	0	0
363	25	1	1				0	0	0	0	0	0	0	0	0	0	0	0	0	1	0	0	0	0	20181112	6.1	622	125000	0	0		
364	25	1	1				0	0	0	0	0	0	0	0	0	0	0	0	0	1	0	0	0	0	20181114	4.4	330	11,700	0	0	0	0
365	22	1	0				0	0	0	0	0	0	0	0	0	0	0	0	0	1	0	0	0	0	20190208	6.3	555	3000	0	0		
366	48	1	1				0	1	1	0	0	0	0	0	0	0	0	0	0	0	1	0	0	0	20190306	5.8	95	13500	0	0		
367	54	1	1				0	0	0	0	0	0	0	0	0	0	0	0	1	1	0	0	0	0	20190612	5.7	459	40,700	0	0	0	0
368	43	1	1				0	1	0	1	0	0	0	0	0	0	0	0	1	1	0	0	0	0	20190717	4.5	113	3330	0	0	0	0

number	age	sex	nation	height	weight	BMI	LLV	underlygir	HTN	DM	CHD	MI	CVA	COPD	CLD	CKD	solid c	hematolo	Charlson	I_INSTI	I_NNRTI	I_PI	I_BIC	I_DTG	ini_lab	ini_wbc	ini_CD4	ini_RNA	ini_HBV	ini_HCV	ini_NRTI_R	ini_INSTI_R
369	28	1	1				0	0	0	0	0	0	0	0	0	0	0	0	0	1	0	0	1	0	20200310	4.5	434	19,300	0	0	0	1
370	37	0	1				0	0	0	0	0	0	0	0	0	0	0	0	0	1	0	0	0	0	20200312	7.9	9	403,000	0	0	0	0
371	48	1	1				0	0	0	0	0	0	0	0	0	0	0	0	0	1	0	0	0	0	20200604	5.5	288	563,000	0	0	0	0
372	23	1	1				0	0	0	0	0	0	0	0	0	0	0	0	0	1	0	0	1	0	20210311	5.7	322	71,900	1	0	0	0
373	44	1	1				0	0	0	0	0	0	0	0	0	0	0	0	0	1	0	0	1	0	20210608	4	96	1060000	0		0	0
374	22	1	1				0	0	0	0	0	0	0	0	0	0	0	0	0	1	0	0	1	0	20220701	2.3	71	1,887,745	0	0	0	0
375	46	1	1				0	1	0	0	0	1	0	0	0	0	0	0	1	1	0	0	0	1	20211028	8.9	156	134000	0	0	0	0
376	23	1	1				0	0	0	0	0	0	0	0	0	0	0	0	0	1	0	0	1	0	20220111	9.9	679	25,468	0	0		
377	30	1	1				0	0	0	0	0	0	0	0	0	0	0	0	0	1	0	0	1	0	20220228	3.5	25	392,895	0	0	0	0
378	32	1	1				0	1	0	0	0	0	1	0	0	0	0	0	1	1	0	0	1	0	20220421	8.2	69	66,271	0	0	0	0
379	33	1	1				0	0	0	0	0	0	0	0	0	0	0	0	0	1	0	0	1	0	20230125	7	422	1,297,671	0	0	0	0
380	30	1	1				0	0	0	0	0	0	0	0	0	0	0	0	0	1	0	0	1	0	20230113	5.5	319	24,639	0	0		
381	30	1	1				0	0	0	0	0	0	0	0	0	0	0	0	0	1	0	0	1	0	20230206	6.9	51	436,329	0	0	0	0

number	ini_PI_R	ini_NNRTI_R	ini_tb	ini_AIDS	ini_OI	LLV_date	LLV_HIV_med	LLV_WBC	LLV_CD4_c	LLV_RNA	L_insti	L_NNRTI	L_PI	L_BIC	L_DTG	final_lab	final_WBC	final_CD4_count	final_RNA	final_INSTI	final_NNRTI	final_PI	final_BIC	final_DTG	death
1	0	1	0	0	0											20171121	4.6	366	0	1	0	0	0	0	0
2			1	1	1											20221215	6.4	452	0	1	0	0	0	0	0
3	1	0	0	0	0											20221027	3.3	391	0	1	0	0	1	0	0
4	0	0	0	1	1											20220818	21.1	293	0	1	0	0	0	1	0
5			0	0	0											20191203	8.7	1504	0	1	0	0	0	0	0
6			0	0	0											20220630	4.5	647	0	1	0	0	1	0	0
7			0	0	0											20221201	5.1	794	0	1	0	0	1	0	0
8			0	0	0											20221020	6.8	811	0	1	0	0	1	0	0
9	0	0	0	0	0											20220512	5.3	524	0	1	0	0	0	0	0
10			0	0	0											20221020	6.7	240	0	1	0	0	1	0	0
11			0	0	0											20220714	7.1	1049	0	1	0	0	0	1	0
12			1	1	1											20220616	8.1	233	0	1	0	0	0	0	0
13			0	0	0											20220526	9.3	662	0	1	0	0	1	0	0
14			0	0	0											20221006	6.7	512	0	1	0	0	1	0	0
15			0	1	1											20220616	7.3	584	0	1	0	0	1	0	0
16			0	0	0											20161213	5.7	358	0	1	0	0	0	0	0
17			0	1	0	20210429	INSTI-based	7.3	773	101	1	0	0	1	0	20221110	5.6	809	65	1	0	0	1	0	0
18			0	1	1											20210709	6.5	885	0	1	0	0	1	0	0
19	0	0	0	0	0											20221229	5.9	1040	0	1	0	0	1	0	0
20			0	0	0											20170221	14.5	591	0	1	0	0	0	0	1
21			0	0	0											20220414	5.4	436	0	1	0	0	0	0	0
22			0	0	0											20220923	14.4	301	0	1	0	0	1	0	0
23			0	1	1											20160419	5.6	504	0	1	0	0	0	0	0
24			0	0	0											20221103	2.6	442	0	1	0	0	0	0	0
25			0	0	0											20220915	6.9	897	0	1	0	0	1	0	0
26			0	0	0											20220922	4.9	873	0	1	0	0	0	1	0
27	0	0	0	1	1											20221222	3.8	252	0	1	0	0	1	0	0
28			0	0	0											20220818	6.6	1048	0	1	0	0	0	1	0
29			0	1	0											20221027	4.8	291	0	0	0	1	0	0	0
30			0	0	0											20160614	4.7	580	0	1	0	0	0	0	0
31			0	1	1											20220407	7.4	692	0	1	0	0	1	0	0
32			0	0	0											20220616	6.1	1297	0	1	0	0	1	0	0
33			0	1	1											20220913	11.4	612	40	1	0	0	1	0	0
34			1	1	1											20141125	5.6	238	0	0	0	1	0	0	0
35			0	0	0											20220707	4.4	231	0	0	0	1	0	0	0
36			0	0	0											20220908	6.2	461	0	1	0	0	1	0	0
37			0	0	0											20220812	10.2	1031	0	1	0	0	1	0	0
38			0	0	1											20220929	6.6	715	0	1	0	0	0	0	0
39			0	0	0											20220804	3.5	609	0	1	0	0	0	1	0
40			1	1	1											20220908	7.5	657	0	1	0	0	0	1	0
41			0	0	0											20220512	8.5	920	0	1	0	0	1	0	0
42			0	0	0											20220603	4.9	570	0	1	0	0	1	0	0
43			0	1	1											20180313	4.3	393	0	1	0	0	0	0	0
44			0	0	0											20220714	7.8	706	0	1	0	0	0	0	0
45			0	0	1											20220915	5	413	0	1	0	0	0	1	0
46			1	1	1											20170110	3.7	448	0	1	0	0	0	0	0

number	ini_PI_R	ini_NNRTI_R	ini_tb	ini_AIDS	ini_OI	LLV_date	LLV_HIV_med	LLV_WBC	LLV_CD4_c	LLV_RNA	L_insti	L_NNRTI	L_PI	L_BIC	L_DTG	final_lab	final_WBC	final_CD4_count	final_RNA	final_INSTI	final_NNRTI	final_PI	final_BIC	final_DTG	death
47	1	0	0	0	0	20200309	INSTI-based	4.9	571	46	1	0	0	1	0	20221020	4.7	615	23	1	0	0	1	0	0
48			0	0	0	20170808	INSTI-based	4.9	688	191	1	0	0	0	0	20210128	5.7	635	0	1	0	0	1	0	0
49			0	1	1											20220721	4.9	443	0	1	0	0	1	0	0
50			0	1	1											20221208	4.6	418	0	1	0	0	1	0	0
51			0	0	0											20221110	3.9	705	0	1	0	0	0	0	0
52			0	1	1											20221222	3.7	243	0	1	0	0	0	1	0
53			0	1	0											20170502	7	299	0	1	0	0	0	0	0
54			0	0	0											20221118	5.6	903	0	1	0	0	1	0	0
55			0	0	0											20220804	6.3	340	0	1	0	0	1	0	0
56			0	0	0											20221006	5.4	385	0	0	0	1	0	0	0
57			0	0	0											20220603	6.2	354	0	1	0	0	0	1	0
58	0	0	0	0	0											20221222	6.5	968	0	1	0	0	1	0	0
59			0	0	0											20220721	5.8	570	0	0	0	1	0	0	0
60			0	0	0											20220519	8.8	556	0	1	0	0	1	0	0
61			0	0	0											20150608	4.6	321	0	1	0	0	0	0	0
62			0	0	0											20180410	7.2	683	0	1	0	0	0	0	0
63			0	0	0											20220901	4	785	0	1	0	0	1	0	0
64			0	0	0	20150414	INSTI-based	5.3	290	59	1	0	0	0	0	20220519	5.6	487	0	1	0	0	1	0	0
65			0	0	0											20220707	5.3	592	0	0	0	1	0	0	0
66			0	0	0											20220519	9.4	1182	0	1	0	0	1	0	0
67			0	0	0											20210218	6.4	811	0	0	0	1	0	0	0
68			0	1	1											20191105	10	331	0	1	0	0	0	0	0
69			0	1	1											20221201	5.7	817	0	1	0	0	1	0	0
70			0	1	1											20220804	5.2	602	0	1	0	0	1	0	0
71			0	0	0											20221027	10.4	722	0	1	0	0	0	1	0
72	0	0	0	0	0											20221020	5.9	357	0	0	0	1	0	0	0
73			0	0	0											20220609	7.2	522	0	1	0	0	1	0	0
74			0	0	0											20221027	4.9	1108	0	1	0	0	1	0	0
75			0	0	0											20221229	6.6	1072	0	1	0	0	1	0	0
76			0	0	0											20221110	7.9	423	0	1	0	0	1	0	0
77			0	0	0											20220929	4.1	352	0	1	0	0	1	0	0
78			0	1	1											20221110	6	545	0	0	0	1	0	0	0
79			0	0	0											20150608	4.9	497	0	1	0	0	0	0	0
80			0	0	0											20220224	6.5	795	0	0	0	1	0	0	0
81			0	1	0											20221229	10.4	316	0	1	0	0	0	0	0
82			0	0	0											20221222	7	1659	0	1	0	0	0	1	0
83			0	0	0											20221020	4.9	498	0	1	0	0	1	0	0
84			1	0	0											20221110	11.4	1469	0	1	0	0	1	0	0
85			0	0	0											20221103	6.1	672	0	1	0	0	1	0	0
86	1	0	0	0	0											20221020	5.9	609	0	1	0	0	1	0	0
87			0	0	0											20221110	5.9	1257	0	1	0	0	1	0	0
88			0	0	0											20220818	5.8	516	0	1	0	0	1	0	0
89			0	1	1											20220317	8.1	784	0	1	0	0	1	0	0
90			0	0	0											20170110	5.9	298	0	1	0	0	0	0	0
91			0	0	0											20190521	5	1000	0	1	0	0	1	0	0
92			0	0	0											20220210	6.3	506	0	1	0	0	1	0	0

number	ini_PI_R	ini_NNRTI_R	ini_tb	ini_AIDS	ini_OI	LLV_date	LLV_HIV_med	LLV_WBC	LLV_CD4_c	LLV_RNA	L_insti	L_NNRTI	L_PI	L_BIC	L_DTG	final_lab	final_WBC	final_CD4_count	final_RNA	final_INSTI	final_NNRTI	final_PI	final_BIC	final_DTG	death
93			0	1	0											20160126	6.9	197	0	1	0	0	0	0	0
94			0	0	0											20210916	7	1052	0	1	0	0	1	0	0
95			0	0	0											20220915	6.1	802	0	1	0	0	1	0	0
96			0	0	0											20160322	3.7	565	0	1	0	0	0	0	0
97			0	0	0											20180116	3.4	649	0	1	0	0	0	0	0
98			0	0	0											20221013	7	961	0	1	0	0	0	1	0
99	0	0	0	0	0											20221117	7.1	706	0	1	0	0	0	0	0
100	0	0	0	0	0											20221222	7.9	956	0	1	0	0	0	1	0
101			0	0	1											20220623	10.3	1852	0	1	0	0	1	0	0
102			0	0	0											20220908	6.6	421	0	1	0	0	1	0	0
103			0	0	0											20160223	6.5	320	0	1	0	0	0	0	0
104			0	0	0											20220929	4.6	473	0	1	0	0	1	0	0
105			0	0	0											20220519	5.6	919	0	1	0	0	1	0	0
106	1	1	0	1	1	20160610	PI-based	5.6	126	158						20220506	7.8	412	78	1	0	0	0	0	0
107			0	0	0											20161018	4.8	414	0	1	0	0	0	0	0
108	0	0	0	1	1											20221216	5	412	0	1	0	0	0	0	0
109			0	0	0											20221020	5.6	771	0	1	0	0	1	0	0
110			0	0	0											20220721	6.5	1009	0	1	0	0	1	0	0
111	1	0	0	0	0											20221229	7	1654	0	1	0	0	1	0	0
112			0	0	0											20221103	5.4	463	0	1	0	0	1	0	0
113	1	0	0	0	0											20160726	8.9	456	0	1	0	0	0	0	0
114	1	0	0	1	1											20181106	6.5	700	0	1	0	0	0	0	0
115			0	0	0											20220714	8.2	877	0	1	0	0	0	1	0
116	0	0	0	0	0											20220922	8.3	1643	0	1	0	0	1	0	0
117	1	0	0	0	0											20220407	4	678	0	1	0	0	1	0	0
118	1	1	0	0	0											20220915	6.4	1088	0	1	0	0	1	0	0
119	1	1	0	0	0											20190129	4.7	872	0	1	0	0	0	0	0
120	1	1	0	0	0	20170110	PI-based	5.4	269	130	0	0	1	0	0	20210422	6.6	302	0	1	0	0	0	0	0
121	1	0	1	1	1											20220623	7.1	768	0	1	0	0	1	0	0
122	0	0	0	0	0											20221201	7.3	1403	0	1	0	0	1	0	0
123			0	0	0											20221124	7.9	684	0	1	0	0	1	0	0
124			0	0	0											20221222	6.6	605	0	1	0	0	1	0	0
125	0	0	0	1	1											20220825	7.3	332	0	1	0	0	1	0	0
126			0	0	0	20201028	INSTI-based	6	597	117	1	0	0	0	0	20221006	5.6	425	0	1	0	0	0	1	0
127			0	0	0											20220616	9.9	1256	0	1	0	0	1	0	0
128			0	1	1											20220811	6	527	0	1	0	0	1	0	0
129			0	0	0											20221027	5.8	598	0	1	0	0	0	1	0
130			0	0	0											20221013	5.4	523	0	1	0	0	1	0	0
131	0	1	0	1	1											20221110	4.3	529	0	1	0	0	1	0	0
132			0	0	0											20221103	6.6	731	0	1	0	0	0	1	0
133	0	0	0	0	0											20180327	8.5	1928	0	1	0	0	0	0	0
134	0	0	0	1	1											20220922	4.7	662	0	1	0	0	1	0	0
135			0	0	0											20221013	5.6	938	0	1	0	0	1	0	0
136			1	1	1											20221215	3.8	281	0	1	0	0	1	0	0
137			0	0	0											20221209	4.2	337	0	1	0	0	1	0	0
138	0	0	0	0	0											20221020	7.1	1006	0	1	0	0	1	0	0

number	ini_PI_R	ini_NNRTI_R	ini_tb	ini_AIDS	ini_OI	LLV_date	LLV_HIV_med	LLV_WBC	LLV_CD4_c	LLV_RNA	L_insti	L_NNRTI	L_PI	L_BIC	L_DTG	final_lab	final_WBC	final_CD4_count	final_RNA	final_INSTI	final_NNRTI	final_PI	final_BIC	final_DTG	death
139	0	0	1	1	1											20221027	7.8	786	0	1	0	0	1	0	0
140			1	1	1											20211125	6.3	608	0	1	0	0	0	1	0
141			0	0	0											20171024	7.9	791	0	1	0	0	0	0	0
142			0	0	0											20221124	6.2	1267	0	1	0	0	1	0	0
143	0	0	0	0	0											20220930	4.5	386	0	1	0	0	1	0	0
144			0	0	0											20220707	7.4	1233	0	1	0	0	0	0	0
145			0	0	0											20221201	3.9	665	0	1	0	0	0	1	0
146			1	1	1											20220714	4.8	519	0	1	0	0	1	0	0
147	0	0	0	0	0											20180814	5.8	694	0	1	0	0	0	0	0
148	0	0	0	0	0											20220512	8.2	1109	0	1	0	0	0	1	0
149	1	0	0	0	0											20221110	4.3	297	86	1	0	0	1	0	0
150	1	0	0	0	0											20221215	6.7	439	0	1	0	0	1	0	0
151	0	0	0	0	0											20221103	7.6	786	0	1	0	0	1	0	0
152	0	0	0	0	0											20221222	6.7	1131	0	1	0	0	1	0	0
153	0	0	0	0	0											20220811	6.5	611	0	1	0	0	1	0	0
154	1	0	0	0	0											20221215	3.6	405	0	1	0	0	0	0	0
155	0	0	0	1	1											20220224	5.2	375	0	1	0	0	1	0	0
156			0	0	0											20221020	4.6	689	0	1	0	0	1	0	0
157	0	1	0	1	1											20221103	5.5	616	0	1	0	0	0	0	0
158	0	0	0	0	0											20220526	5.7	1167	0	1	0	0	1	0	0
159	0	0	0	0	0											20191119	6.9	415	0	1	0	0	0	0	0
160			0	0	0	20220811	INSTI-based	5.4	776	51	1	0	0	1	0	20221117	6.1	873	44	1	0	0	1	0	0
161	0	0	0	0	0											20220929	5	706	0	1	0	0	1	0	0
162	0	0	0	0	0											20220630	6.5	1083	0	1	0	0	1	0	0
163	0	0	0	0	0											20220922	6.4	1022	0	1	0	0	1	0	0
164	0	0	0	0	0											20190604	4.8	450	0	1	0	0	0	0	0
165	0	0	0	0	0											20221103	6.3	956	0	1	0	0	0	1	0
166	0	0	0	0	0											20191008	5.9	577	0	1	0	0	0	0	0
167	0	0	0	0	0											20221222	9.3	1219	0	1	0	0	1	0	0
168	0	0	0	0	0											20221027	7.9	740	0	1	0	0	1	0	0
169			1	1	1											20220707	5.1	528	0	1	0	0	1	0	0
170			0	0	0											20210812	6.4	765	0	1	0	0	1	0	0
171	1	0	0	0	0											20201126	14	606	27	1	0	0	1	0	0
172	0	0	0	1	1											20221229	6.1	276	0	1	0	0	0	1	0
173	0	0	0	0	0											20220922	5.6	647	0	1	0	0	1	0	0
174	1	0	0	0	0											20221125	4.7	719	0	1	0	0	0	1	0
175	0	0	0	0	0											20220728	4.8	939	0	1	0	0	0	1	0
176			0	0	0											20190604	3.3	450	0	1	0	0	0	0	0
177			0	1	1											20220506	5.5	303	0	1	0	0	0	0	0
178			0	0	0											20220915	5.2	1052	0	1	0	0	1	0	0
179	0	0	0	0	0											20210819	6.5	755	0	1	0	0	0	1	0
180	0	0	1	1	1											20220721	6.6	319	0	1	0	0	1	0	0
181			0	0	0											20220915	6.3	636	0	1	0	0	1	0	0
182	1	0	0	0	0											20230105	5.7	769	0	1	0	0	1	0	0
183	1	0	0	0	0											20220630	6.9	618	0	1	0	0	0	1	0
184	1	0	1	1	1											20221201	5.8	507	0	1	0	0	1	0	0

number	ini_PI_R	ini_NNRTI_R	ini_tb	ini_AIDS	ini_OI	LLV_date	LLV_HIV_med	LLV_WBC	LLV_CD4_c	LLV_RNA	L_insti	L_NNRTI	L_PI	L_BIC	L_DTG	final_lab	final_WBC	final_CD4_count	final_RNA	final_INSTI	final_NNRTI	final_PI	final_BIC	final_DTG	death
185	0	0	0	0	0											20220707	5.4	971	0	1	0	0	0	0	0
186	0	0	0	0	0	20210429	INSTI-based	4.1	374	101	1	0	0	1	0	20220819	3.7	254	24	1	0	0	1	0	0
187	0	0	0	0	0											20221013	6	872	0	1	0	0	1	0	0
188			0	0	0											20230105	5.8	581	0	1	0	0	1	0	0
189			0	0	0											20221208	5.3	703	0	1	0	0	1	0	0
190	1	1	0	0	0											20220915	4.6	656	0	1	0	0	0	1	0
191	0	1	0	0	0											20220512	7	929	0	1	0	0	0	1	0
192			0	0	0											20220707	3.6	382	0	1	0	0	1	0	0
193	1	0	0	0	0											20220728	8.8	495	0	1	0	0	0	1	0
194	1	0	0	0	0											20221201	3.7	624	0	1	0	0	0	0	0
195			0	0	0											20220915	5.9	522	0	1	0	0	1	0	0
196			0	0	0											20220922	3.9	595	0	1	0	0	1	0	0
197	0	0	0	1	1											20220804	5.8	602	0	1	0	0	1	0	0
198			0	1	1											20221020	4.1	228	0	1	0	0	1	0	0
199			0	0	0											20221021	7.3	870	121	1	0	0	1	0	0
200	0	0	0	0	0											20221202	6.7	1483	0	1	0	0	0	1	0
201	1	1	0	0	0											20220714	13.2	1038	0	1	0	0	1	0	0
202			0	0	0											20220616	5.2	479	0	1	0	0	1	0	0
203	0	0	0	0	0	20201015	INSTI-based	7.1	1125	75	1	0	0	1	0	20220825	9.6	796	102	1	0	0	1	0	0
204	1	0	0	0	0											20220908	7.1	1077	0	1	0	0	1	0	0
205	0	1	0	0	0											20221222	4.3	377	0	1	0	0	1	0	0
206	0	0	0	0	0	20211111	INSTI-based	7	528	70	1	0	0	1	0	20221027	5.8	520	0	1	0	0	1	0	0
207	0	0	0	0	0											20221103	4.6	801	0	1	0	0	1	0	0
208	0	0	0	0	0											20220901	10.7	674	0	1	0	0	1	0	0
209	1	1	0	0	0											20221208	6.1	921	0	1	0	0	1	0	0
210	1	1	0	0	0											20221013	5.9	713	0	1	0	0	1	0	0
211	1	0	0	0	0											20221222	5.2	454	0	1	0	0	1	0	0
212	0	0	0	0	0											20221118	9.4	882	0	1	0	0	1	0	0
213	1	1	0	0	0											20200205	8.2	79	0	1	0	0	1	0	0
214			0	0	0											20221013	5.5	444	0	1	0	0	1	0	0
215	0	0	0	0	0											20220428	9.7	866	0	0	0	1	0	0	0
216	1	0	0	0	0											20220930	8.8	935	0	1	0	0	0	1	0
217	0	0	0	0	0											20221124	11.4	258	0	1	0	0	0	1	0
218			0	0	0											20221117	4.7	682	0	1	0	0	0	1	0
219	0	0	0	0	0											20220908	5.7	455	0	1	0	0	1	0	0
220			0	0	0											20220915	5.7	420	0	1	0	0	1	0	0
221			0	0	0											20220901	8.9	1060	0	1	0	0	0	1	0
222	0	0	0	0	0											20221201	8.2	748	0	1	0	0	1	0	0
223	1	1	0	0	0											20211125	4.2	288	0	0	0	1	0	0	0
224	0	0	0	0	0											20220512	7.1	498	0	1	0	0	0	1	0
225	0	1	0	1	1	20200408	INSTI-based	9.5	221	85	1	0	0	1	0	20220811	8.7	432	0	1	0	0	1	0	0
226	0	0	0	0	0											20220825	12	389	0	1	0	0	0	1	0
227			0	1	1											20221006	6.5	263	0	1	0	0	1	0	1
228			0	0	0											20201118	9.6	884	0	1	0	0	1	0	0
229	0	0	0	0	0											20221007	7.4	818	26	1	0	0	1	0	0
230	0	0	0	0	0											20220818	3.4	358	0	1	0	0	1	0	0

number	ini_PI_R	ini_NNRTI_R	ini_tb	ini_AIDS	ini_OI	LLV_date	LLV_HIV_med	LLV_WBC	LLV_CD4_c	LLV_RNA	L_insti	L_NNRTI	L_PI	L_BIC	L_DTG	final_lab	final_WBC	final_CD4_count	final_RNA	final_INSTI	final_NNRTI	final_PI	final_BIC	final_DTG	death
231			0	0	0											20220908	10.6	1223	0	1	0	0	1	0	0
232			0	0	0											20210114	6	562	0	1	0	0	1	0	0
233	1	1	0	0	0											20220818	4.7	785	0	1	0	0	1	0	0
234			0	0	0											20221215	7.3	832	0	1	0	0	1	0	0
235	0	0	1	0	0											20220819	8.4	673	0	1	0	0	0	1	0
236	0	1	0	0	0											20220421	6.7	384	0	1	0	0	1	0	0
237			0	0	0											20221215	5.5	595	0	1	0	0	1	0	0
238	0	0	0	0	0											20220623	4.2	635	0	1	0	0	1	0	0
239	0	0	0	0	0											20221110	6	754	0	1	0	0	1	0	0
240			0	0	0											20220929	5	534	0	1	0	0	1	0	0
241			0	0	0											20221125	5.1	466	30	1	0	0	1	0	0
242			0	0	0											20221215	5.7	521	0	1	0	0	1	0	0
243			0	0	0											20220707	7.1	399	0	1	0	0	1	0	0
244			0	0	0											20220922	5.7	250	0	1	0	0	0	1	0
245	0	0	0	0	0	20210531	INSTI-based	5.3	308	98	1	0	0	1	0	20221201	5.4	366	78	1	0	0	1	0	0
246	1	0	0	1	1	20220127	INSTI-based	8.1	398	88	1	0	0	1	0	20220804	6.3	367	43	1	0	0	1	0	0
247	1	0	0	0	0											20221013	3.5	281	0	1	0	0	1	0	0
248	0	0	0	0	0											20221124	5.7	466	0	1	0	0	1	0	0
249	0	0	0	0	0											20220609	5.8	512	0	1	0	0	1	0	0
250	1	0	0	0	0											20220630	5.1	623	0	1	0	0	1	0	0
251	0	0	0	0	0											20220707	4.5	533	0	1	0	0	1	0	0
252	0	0	0	0	0											20220804	7.1	750	0	1	0	0	1	0	0
253			0	0	0											20221110	9.6	431	0	1	0	0	1	0	0
254	0	0	0	0	0											20220922	4.7	640	0	1	0	0	1	0	0
255	0	0	0	0	0											20220616	5.9	683	0	1	0	0	1	0	0
256	1	0	0	0	0											20220804	6.3	560	0	1	0	0	0	1	0
257	0	0	0	0	0											20221027	9.7	745	24	1	0	0	1	0	0
258			0	0	0											20220707	5.4	783	0	1	0	0	1	0	0
259	0	0	0	0	0											20220728	9.1	822	0	1	0	0	1	0	0
260	0	0	0	0	0											20220609	5.9	645	0	1	0	0	0	1	0
261			0	0	0											20220623	11.5	938	43	1	0	0	1	0	0
262			0	0	0											20221201	4.5	737	0	1	0	0	1	0	0
263			0	0	0											20221103	4.8	446	0	1	0	0	0	0	0
264			0	0	0											20221110	6.4	196	0	1	0	0	0	0	0
265			0	0	0											20221117	4.4	616	0	1	0	0	1	0	0
266	1	0	0	0	0											20220609	5	211	0	1	0	0	1	0	0
267			0	1	0											20221103	3.5	108	0	1	0	0	1	0	0
268	0	1	0	0	0											20221020	9.6	916	0	1	0	0	0	1	0
269	0	0	0	1	1											20221027	7	85	0	1	0	0	1	0	0
270	0	0	0	0	0											20220603	6.8	449	0	1	0	0	1	0	0
271			0	1	1											20221117	5.1	267	63	1	0	0	0	1	0
272	1	0	0	0	0											20221006	5.9	1001	30	1	0	0	1	0	0
273			0	0	0											20220630	9	507	0	1	0	0	1	0	0
274			0	0	0											20220707	5	484	0	1	0	0	0	1	0
275	1	0	0	0	0											20220623	4.6	552	0	1	0	0	1	0	0
276	0	0	0	0	0											20221007	7.3	592	67	1	0	0	1	0	0

number	ini_PI_R	ini_NNRTI_R	ini_tb	ini_AIDS	ini_OI	LLV_date	LLV_HIV_med	LLV_WBC	LLV_CD4_c	LLV_RNA	L_insti	L_NNRTI	L_PI	L_BIC	L_DTG	final_lab	final_WBC	final_CD4_count	final_RNA	final_INSTI	final_NNRTI	final_PI	final_BIC	final_DTG	death
277	1	0	0	0	0											20220811	6.2	423	0	1	0	0	1	0	0
278	0	1	0	1	1											20221222	5.7	209	0	1	0	0	0	1	0
279	0	0	0	0	0											20221229	5	609	0	1	0	0	0	1	0
280	0	0	0	0	0											20220804	8.5	782	0	1	0	0	0	1	0
281	0	0	0	1	1											20221222	7.7	303	0	1	0	0	1	0	0
282	0	0	0	0	0											20220922	5.3	763	0	1	0	0	0	1	0
283			0	0	0											20221208	4.9	751	0	1	0	0	0	1	0
284	0	0	0	0	0											20221013	8.3	1139	31	1	0	0	1	0	0
285	1	0	0	0	0											20231214	5.6	999.2304	0	1	0	0	0	0	0
286			1	1	1											20191028	5.3	154.2777	0	0	1	0	0	0	0
287			0	0	0											20230918	8	779.24	155	1	0	0	1	0	0
288	0	0	0	0	0											20231020	7	847.665	25	1	0	0	0	1	0
289	0	0	0	0	0											20231205	4.2	758.814	0	1	0	0	0	1	0
290			0	0	0											20230926	6.9	678.132	0	1	0	0	0	1	0
291			0	0	0											20231205	14.3	1086.228	0	1	0	0	0	1	0
292			0	0	0											20230731	6.8	810.7776	0	1	0	0	0	1	0
293			0	0	0											20231016	5.6	417.7152	0	1	0	0	0	1	0
294	0	0	0	0	0											20231017	6	1061.91	0	1	0	0	0	1	0
295			0	0	0											20160523	5.4	738.05904	0	1	0	0	0	0	0
296	0	0	0	0	0											20230918	4.6	622.564	0	1	0	0	1	0	0
297			0	0	0											20230215	9.5	1112.412	0	1	0	0	1	0	0
298	0	1	0	1	1											20231219	4.9	212.9148	21	1	0	0	1	0	0
299			0	0	0											20230926	5.7	487.9656	0	1	0	0	0	1	0
300			0	0	0											20221006	21.7	419.7648	0		1	0	0	0	1
301			0	0	0											20231205	5.8	902.4858	0	1	0	0	1	0	0
302			0	0	0											20231031	6.1	1102.941	0	1	0	0	0	1	0
303			0	0	0											20200715	5.9	723.7176	0	1	0	0	0	0	0
304			0	0	0											20231108	8.2	926.477	0	1	0	0	0	0	0
305	0	0	0	0	0											20230818	9.3	756.4992	0	1	0	0	1	0	0
306	0	0	0	0	0											20231212	7.8	658.7568	0	1	0	0	0	1	0
307			0	0	0											20230404	11.1	506.7039	0	1	0	0	0	0	0
308			0	0	0											20231127	9.2	694.1308	0	1	0	0	1	0	0
309			0	1	1											20230728	7.6	1283.0624	0	1	0	0	1	0	0
310			0	0	0											20230925	6.3	1002.456	0	1	0	0	1	0	0
311			0	0	0											20231120	4.9	697.1034	0	1	0	0	0	1	0
312			0	0	0											20160428	6.7	874.35	0		0	1	0	0	0
313			1	1	1											20231208	5.8	432.564	0	1	0	0	0	1	0
314			0	0	0											20230331	8.9	907.088	0	1	0	0	1	0	0
315			0	1	1											20231110	8.5	858.245	0	1	0	1	0	0	0
316			0	1	1											20230919	4.8	150.9984	0	1	0	0	1	0	0
317			0	0	0											20231228	5.8	694.6602	0	1	0	0	1	0	0
318			0	0	0											20231113	6.8	1241.136	0	1	0	0	1	0	0
319			0	1	1											20231120	4	196.384	0	1	0	0	1	0	0
320			0	0	0											20230925	8.2	983.1718	0	1	0	0	0	1	0
321			0	1	1											20231005	2	336.96	0	1	0	0	0	1	0
322			0	0	0											20231127	6.7	539.0418	0	1	0	0	1	0	0

number	ini_PI_R	ini_NNRTI_R	ini_tb	ini_AIDS	ini_OI	LLV_date	LLV_HIV_med	LLV_WBC	LLV_CD4_c	LLV_RNA	L_insti	L_NNRTI	L_PI	L_BIC	L_DTG	final_lab	final_WBC	final_CD4_count	final_RNA	final_INSTI	final_NNRTI	final_PI	final_BIC	final_DTG	death
323			0	1	1											20221004	4	547.976	0	1	0	0	1	0	1
324	0	0	0	0	0											20231212	10.1	795.6578	0	1	0	0	0	1	0
325			0	0	0											20230725	7.8	749.5488	0	1	0	0	0	1	0
326	0	1	0	0	0											20230818	8.4	628.5048	0	1	0	0	0	1	0
327			0	0	0											20231023	5.8	728.8164	0	1	0	0	0	1	0
328			0	0	0											20230921	6.5	724.152	0	1	0	0	1	0	0
329			1	1	1											20231222	5.8	330.8088	0	1	0	0	0	1	0
330			0	0	0											20230817	6.4	1006.624	0	1	0	0	0	1	0
331			0	0	0											20230905	9.2	500.9216	0	1	0	0	1	0	0
332			0	0	0											20230810	9.9	441.7578	0	1	0	1	0	0	0
333			0	1	1											20230821	8.2	1084.368	0	1	0	0	1	0	0
334			0	0	0											20231211	5.8	1056.9108	0	1	0	0	1	0	0
335			0	0	0											20231121	7.5	819.735	0	1	0	0	1	0	0
336	0	0	0	0	0											20230921	5.2	426.5976	0	1	0	0	1	0	0
337			0	1	1											20151221	4.4	286.528	0	1	0	0	0	0	0
338			0	0	0											20231116	5.7	805.752	0	1	0	0	1	0	0
339			1	1	1											20161013	4.2	580.76676	0	1	0	0	0	0	0
340			0	0	0											20231124	6	533.76	0	1	0	0	0	1	0
341			0	0	0											20230628	7.3	694.6461	0	1	0	0	0	1	0
342			0	0	0											20230807	8.3	728.574	0	1	0	0	0	1	0
343			0	0	0											20231205	4.3	320.9907	0	1	0	0	1	0	0
344	0	0	0	1	1											20231206	4.9	652.2096	0	1	0	0	1	0	0
345			0	1	1											20231120	5.3	493.695	0	1	0	0	0	1	0
346			0	0	0											20230823	5.3	541.554	0	1	0	0	1	0	0
347			0	1	0											20230810	4.8	329.4144	0	1	0	0	0	1	0
348			0	0	0											20201116	4	994.896	0	1	0	0	1	0	0
349	0	0	0	0	0											20231004	9.2	1089.28	0	1	0	0	0	1	0
350			0	0	0											20231219	10.2	300.543	0	1	0	0	1	0	0
351			0	0	0											20231128	10.1	1146.1884	0	1	0	0	0	1	0
352			0	1	0	20161114	PI-based			76.8			1			20231212	5.3	674.2872	0	1	0	0	1	0	0
353			0	1	1											20230630	8.2	713.4738	0	1	0	0	1	0	0
354	0	0	0	1	1											20231219	7.8	424.4448	0	1	0	0	0	1	0
355			0	1	1											20231129	5	196.52	0	1	0	0	1	0	0
356	0	0	0	0	0											20231122	3.8	436.8024	0	1	0	0	1	0	0
357			0	0	0											20231010	5.7	231.9786	0	1	0	0	1	0	0
358			0	1	1											20180212	6.3	319.08114	24.3	1	0	0	0	0	0
359			0	0	0											20180129	8.7	976.55064	0	1	0	0	0	0	0
360	0	1	0	0	0	20201204	INSTI-based		345	43.2	1			1		20230328	8.7	519.042	0	1	0	0	1	0	0
361			0	0	0											20230627	6.7	1143.5292	0	1	0	0	1	0	0
362	0	0	0	0	0											20231018	4	273.6	0	1	0	0	0	1	0
363			0	0	0											20230724	4.6	556.6414	0	1	0	0	0	0	0
364	0	1	0	0	0											20231219	6.3	957.2094	0	1	0	0	0	1	0
365			0	0	0											20230926	4.8	705.0048	0	1	0	0	0	1	0
366			1	1	1											20230912	6.7	523.136	0	1	0	0	1	0	0
367	0	0	0	0	0											20210706	8.8	1284.976	0	1	0	0	1	0	0
368	0	0	0	0	0											20231011	7.6	331.056	0	1	0	0	1	0	0

number	ini_PI_R	ini_NNRTI_R	ini_tb	ini_AIDS	ini_OI	LLV_date	LLV_HIV_med	LLV_WBC	LLV_CD4_c	LLV_RNA	L_insti	L_NNRTI	L_PI	L_BIC	L_DTG	final_lab	final_WBC	final_CD4_count	final_RNA	final_INSTI	final_NNRTI	final_PI	final_BIC	final_DTG	death
369	0	0	0	0	0											20220905	5.9	642.3625	0	1	0	0	1	0	0
370	0	0	0	1	1											20231117	5.8	260.9188	0	1	0	0	1	0	0
371	0	0	0	0	0											20250418	6.3	426	28	1	0	0	0	1	0
372	0	0	0	0	0											20211117	5.4	622.485	0	1	0	0	1	0	0
373	0	0	0	1	1											20231226	5.2	415.2928	0	1	0	0	1	0	0
374	0	1	0	1	1											20230714	5.1	470.2914	25	1	0	0	1	0	0
375	0	0	0	0	0											20230809	9.3	591.9264	25	1	0	0	0	1	0
376			0	0	0											20231103	6.3	700.8687	0	1	0	0	1	0	0
377	0	1	0	1	1											20231031	8.8	384.78	0	1	0	0	1	0	0
378	0	0	0	1	1											20231030	5.3	305.704	0	1	0	0	1	0	0
379	0	0	0	0	0											20231107	5	461.7	0	1	0	0	0	1	0
380			0	0	0											20231020	4.2	451.1976	0	1	0	0	1	0	0
381	0	0	0	0	0											20231214	6.6	232.3398	0	1	0	0	1	0	0

number	rebound	final_AIDS	final_OI	final_HIV_R	final_NRTI_R	final_INSTI_R	final_PI_R	final_NNRTI_R
1	0	0	0					
2	0	0	0					
3	0	0	0					
4	0	0	0					
5	0	0	0					
6	0	0	0					
7	0	0	0					
8	0	1	0					
9	0	0	0					
10	0	0	0					
11	0	0	0					
12	0	0	0					
13	0	0	0					
14	0	0	0					
15	0	0	0					
16	0	0	0					
17	0	0	0					
18	0	0	0					
19	0	0	0					
20	0	0	0					
21	0	0	0					
22	0	0	0					
23	0	0	0					
24	0	0	0					
25	0	0	0					
26	0	0	0					
27	0	0	0					
28	0	1	1					
29	0	0	1					
30	0	0	0					
31	0	0	0					
32	0	0	0					
33	0	0	0					
34	0	0	0					
35	0	0	0					
36	0	1	1					
37	0	0	0					
38	0	0	0					
39	0	1	1					
40	1	0	0	1	1	0	1	1
41	0	0	0					
42	0	0	0					
43	0	0	0					
44	0	0	0	1	0	1	1	0
45	0	1	1					
46	0	0	0					

number	rebound	final_AIDS	final_OI	final_HIV_R	final_NRTI_R	final_INSTI_R	final_PI_R	final_NNRTI_R
47	0	0	0					
48	0	0	0					
49	0	0	0					
50	0	0	0					
51	0	0	0					
52	0	1	0					
53	0	0	0					
54	0	0	0					
55	0	0	0					
56	0	0	0					
57	0	0	0					
58	0	0	0					
59	0	0	0					
60	0	0	0					
61	0	0	0					
62	0	0	0					
63	0	0	0					
64	0	0	0					
65	0	0	0					
66	0	0	0					
67	0	0	0					
68	0	0	0					
69	0	0	0					
70	0	0	0					
71	0	0	0					
72	0	0	0					
73	0	0	0					
74	0	0	0					
75	0	0	0					
76	0	0	0					
77	0	0	0					
78	0	0	0					
79	0	0	0					
80	0	0	0					
81	0	0	0					
82	0	0	0					
83	0	0	0					
84	0	0	0					
85	0	0	0					
86	0	0	0					
87	0	0	0					
88	0	0	0					
89	0	0	0					
90	0	0	0					
91	0	0	0					
92	0	0	0					

number	rebound	final_AIDS	final_OI	final_HIV_R	final_NRTI_R	final_INSTI_R	final_PI_R	final_NNRTI_R
93	0	0	0					
94	0	0	0					
95	0	0	0					
96	0	0	0					
97	0	0	0					
98	0	0	0					
99	0	0	0					
100	0	0	0					
101	0	0	0					
102	0	0	0					
103	0	0	0					
104	0	0	0					
105	0	0	0					
106	1	1	1					
107	0	0	0					
108	0	0	0					
109	0	0	0					
110	0	0	0					
111	0	0	0					
112	0	0	0					
113	0	0	0					
114	0	0	0					
115	0	0	0					
116	0	0	0					
117	0	0	0					
118	0	0	0					
119	0	0	0					
120	0	0	0					
121	0	0	0					
122	0	0	0					
123	0	0	0					
124	0	0	0					
125	0	0	0					
126	0	0	0					
127	0	0	0					
128	0	0	0					
129	0	0	0					
130	0	0	0					
131	0	0	0					
132	0	0	0					
133	0	0	0					
134	0	1	1					
135	0	0	0					
136	0	0	0					
137	0	0	0					
138	0	0	0					

number	rebound	final_AIDS	final_OI	final_HIV_R	final_NRTI_R	final_INSTI_R	final_PI_R	final_NNRTI_R
139	0	0	0					
140	0	0	0					
141	0	0	0					
142	0	0	0					
143	0	0	0					
144	0	0	0					
145	0	0	0					
146	0	0	0					
147	0	0	0					
148	0	0	0					
149	0	0	0					
150	0	0	0					
151	0	0	0					
152	0	0	0					
153	0	0	0					
154	0	0	0					
155	0	0	0					
156	0	0	0					
157	0	0	0					
158	0	0	0					
159	0	0	0					
160	0	0	0					
161	0	0	0					
162	0	0	0					
163	0	0	0					
164	0	0	0					
165	0	0	0					
166	0	0	0					
167	0	0	0					
168	0	0	0					
169	0	0	0					
170	0	0	0					
171	0	0	0					
172	0	0	0					
173	0	0	0					
174	0	0	0					
175	0	0	0					
176	0	0	0					
177	0	0	0					
178	0	0	0					
179	0	0	0					
180	0	0	0					
181	0	0	0					
182	0	0	0					
183	0	0	0					
184	0	0	0					

number	rebound	final_AIDS	final_OI	final_HIV_R	final_NRTI_R	final_INSTI_R	final_PI_R	final_NNRTI_R
185	0	0	0					
186	0	0	0					
187	0	0	0					
188	0	0	0					
189	0	0	0					
190	0	0	0					
191	0	0	0					
192	0	0	0					
193	0	0	0					
194	0	0	0					
195	0	0	0					
196	0	0	0					
197	0	0	0					
198	0	0	0					
199	0	0	0					
200	0	0	0					
201	0	0	0					
202	0	0	0					
203	0	0	0					
204	0	0	0					
205	0	0	0					
206	0	0	0					
207	0	0	0					
208	0	0	0					
209	0	0	0					
210	0	0	0					
211	0	0	0					
212	0	0	0					
213	0	0	0					
214	0	0	0					
215	0	0	0					
216	0	0	0					
217	0	0	0					
218	0	0	0					
219	0	0	0					
220	0	0	0					
221	0	0	0					
222	0	0	0					
223	0	0	0					
224	0	0	0					
225	0	0	0					
226	0	0	0					
227	0	0	0					
228	0	0	0					
229	0	0	0					
230	0	0	0					

number	rebound	final_AIDS	final_OI	final_HIV_R	final_NRTI_R	final_INSTI_R	final_PI_R	final_NNRTI_R
231	0	0	0					
232	0	0	0					
233	0	0	0					
234	0	0	0					
235	0	0	0					
236	0	0	0					
237	0	0	0					
238	0	0	0					
239	0	0	0					
240	0	0	0					
241	0	0	0					
242	0	0	0					
243	0	0	0					
244	0	0	0					
245	0	0	0					
246	0	0	0					
247	0	0	0					
248	0	0	0					
249	0	0	0					
250	0	0	0					
251	0	0	0					
252	0	0	0					
253	0	0	0					
254	0	0	0					
255	0	0	0					
256	0	0	0					
257	0	0	0					
258	0	0	0					
259	0	0	0					
260	0	0	0					
261	0	0	0					
262	0	0	0					
263	0	0	0					
264	0	0	0					
265	0	0	0					
266	0	0	0					
267	0	0	0					
268	0	0	0					
269	0	0	0					
270	0	0	0					
271	0	0	0					
272	0	0	0					
273	0	0	0					
274	0	0	0					
275	0	0	0					
276	0	0	0					

number	rebound	final_AIDS	final_OI	final_HIV_R	final_NRTI_R	final_INSTI_R	final_PI_R	final_NNRTI_R
277	0	0	0					
278	0	0	0					
279	0	0	0					
280	0	0	0					
281	0	0	0					
282	0	0	0					
283	0	0	0					
284	0	0	0					
285	0	0	0					
286	0	0	0	0				
287	0	0	0					
288	0	0	0					
289	0	0	0					
290	0	0	0					
291	0	0	0					
292	0	0	0					
293	0	0	0					
294	0	0	0					
295	0	0	0					
296	0	0	0					
297	0	0	0					
298	0	0	0					
299	0	0	0					
300	0	0	0					
301	0	0	0					
302	0	0	0					
303	0	0	0	3				
304	0	0	0					
305	0	0	0					
306	0	0	0					
307	0	0	0					
308	0	0	0					
309	0	0	0					
310	0	0	0					
311	0	0	0					
312	0	0	0					
313	0	0	0					
314	0	0	0					
315	0	0	0					
316	0	0	0					
317	0	0	0					
318	0	0	0					
319	0	0	0					
320	0	0	0					
321	0	0	0					
322	0	1	1					

number	rebound	final_AIDS	final_OI	final_HIV_R	final_NRTI_R	final_INSTI_R	final_PI_R	final_NNRTI_R
323	0	0	0					
324	0	0	0					
325	0	0	0					
326	0	0	0					
327	0	0	0					
328	0	0	0					
329	0	0	0					
330	0	0	0					
331	0	0	0					
332	0	0	0					
333	0	0	0					
334	0	0	0					
335	0	0	0					
336	0	0	0					
337	0	0	0					
338	0	0	0					
339	0	0	0					
340	0	0	0					
341	0	0	0					
342	0	0	0					
343	0	0	0					
344	0	0	0					
345	0	0	0					
346	0	0	0					
347	0	0	0					
348	0	0	0					
349	0	0	0					
350	0	0	0					
351	0	0	0					
352	0	0	0					
353	0	0	0					
354	0	0	0					
355	0	0	0					
356	0	0	0					
357	0	0	0					
358	0	0	0					
359	0	0	0					
360	0	0	0					
361	0	0	0					
362	0	0	0					
363	0	0	0					
364	0	0	0					
365	0	0	0					
366	0	0	0					
367	0	0	0					
368	0	0	0					

number	rebound	final_AIDS	final_OI	final_HIV_R	final_NRTI_R	final_INSTI_R	final_PI_R	final_NNRTI_R
369	0	0	0					
370	0	0	0					
371	0	0	0					
372	0	0	0					
373	0	0	0					
374	0	0	0					
375	0	0	0					
376	0	0	0					
377	0	0	0					
378	0	0	0					
379	0	0	0					
380	0	0	0					
381	0	0	0					
